# Supplementary material for: Microneedle-based sampling of dermal interstitial fluid using a vacuum-assisted skin patch
Source: Cell Rep Phys Sci. 2024 Jun 19;5(6):101975. doi: 10.1016/j.xcrp.2024.101975 (PMC11211974; doi:10.1016/j.xcrp.2024.101975)
Supplement: Document S1. Figures S1–S12 and Tables S1–S3 [file mmc1.pdf]

Cell Reports Physical Science, Volume *5*

Supplemental information

Microneedle-based sampling of dermal interstitial fluid using a vacuum-assisted skin patch

Xue Jiang, Elizabeth C. Wilkirson, Aaron O. Bailey, William K. Russell, and Peter B. Lillehoj

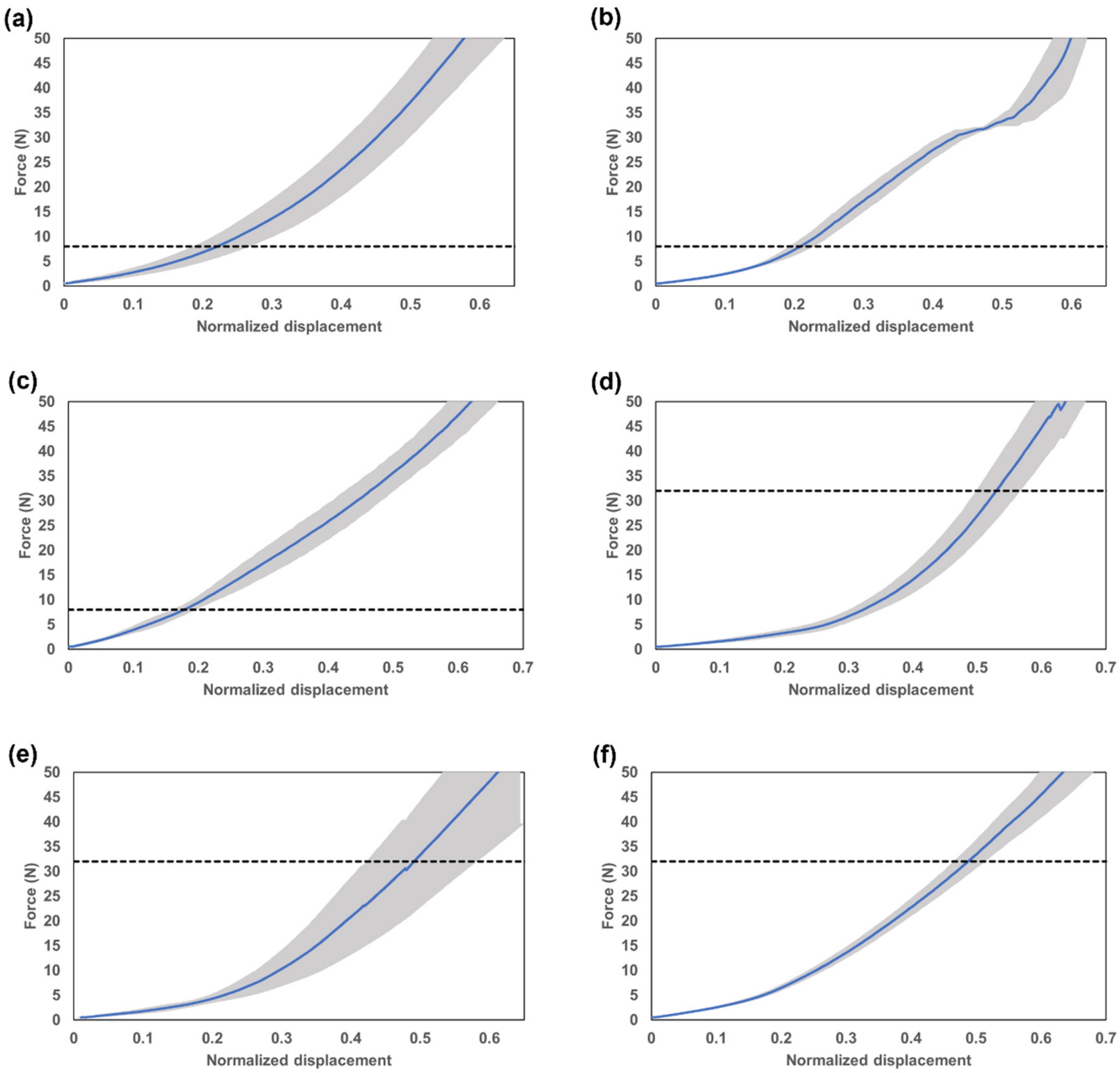

Figure S1. Mechanical strength of MN arrays. Force vs. displacement curves for 10 × 10 MN arrays with needle lengths of (a) 450 μm, (b) 600 μm or (c) 750 μm, and (d) 20 × 20 MN arrays with needle lengths of 450 μm, (e) 600 μm, or (f) 750 μm. Solid lines represent the mean from 3 separate measurements using new MN arrays. Shaded region represents the standard deviation. Horizontal dotted lines represent the force required to penetrate human skin.

Cell Reports
Physical Science

CellPress

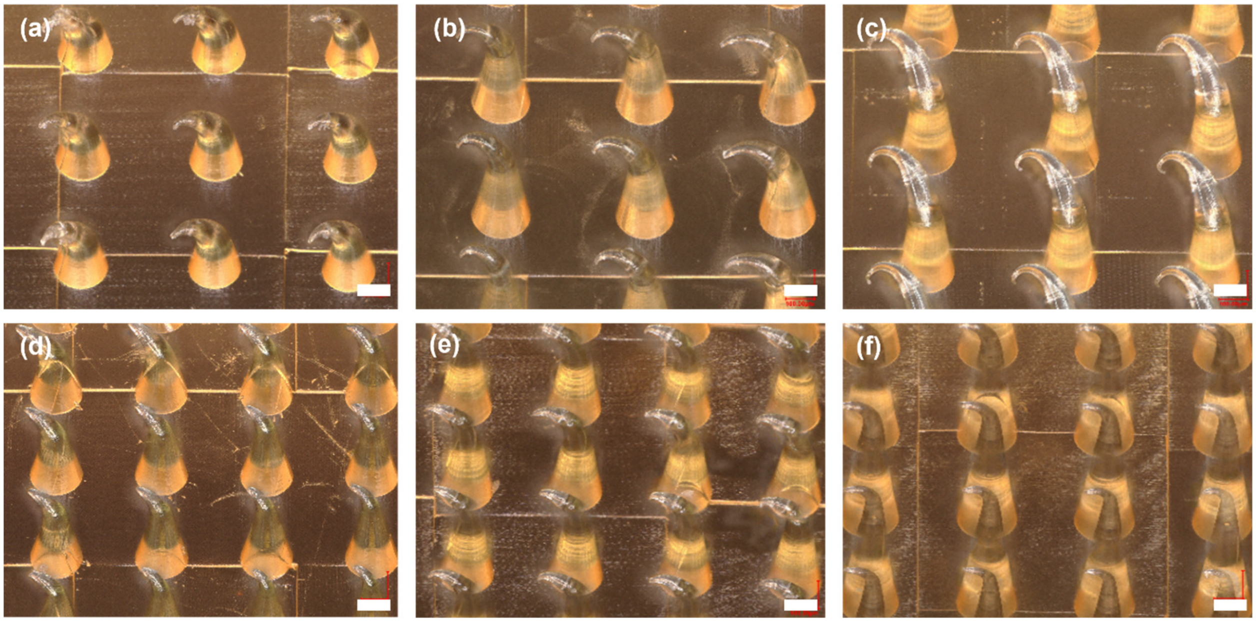

Figure S2. Compression testing of MN arrays at maximum load. Optical micrographs of 10 × 10 MN arrays with needle lengths of (a) 450 μm, (b) 600 μm, or (c) 750 μm, and (d) 20 × 20 MN arrays with needle lengths of 450 μm, (e) 600 μm, or (f) 750 μm. Scale bars, 100 μm.

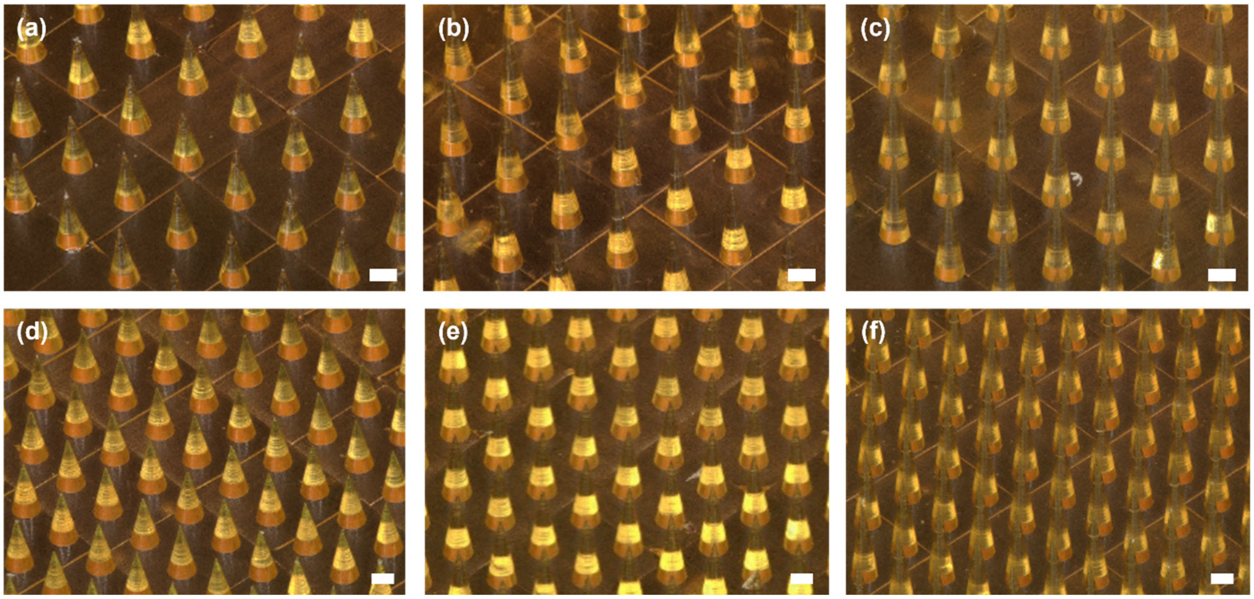

Figure S3. MN integrity after repeated skin penetration. Optical micrographs of 10 × 10 MN arrays with needle lengths of (a) 450 μm, (b) 600 μm, or (c) 750 μm after 36 insertions in porcine skin, and (d) 20 × 20 MN arrays with needle lengths of 450 μm, (e) 600 μm, or (f) 750 μm after 12 insertions. Scale bars, 1000 μm. Insets show close-up views of the MNs. Scale bars, 100 μm.

Cell Reports
Physical Science

CellPress

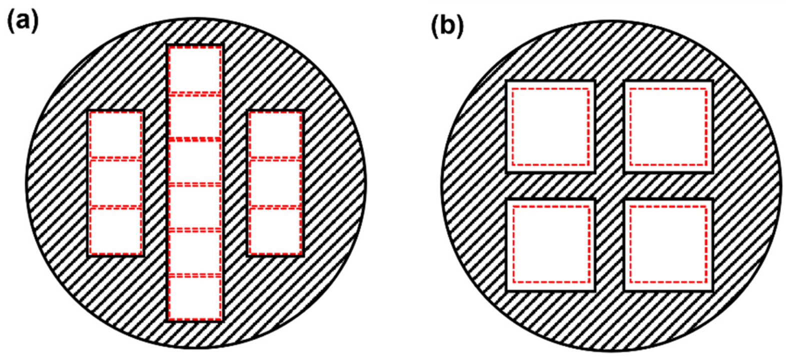

Figure S4. Designs of the skin patch sticker. MN array application sites outlined in red for the (a) 10 × 10 MN array and (b) 20 × 20 MN array.

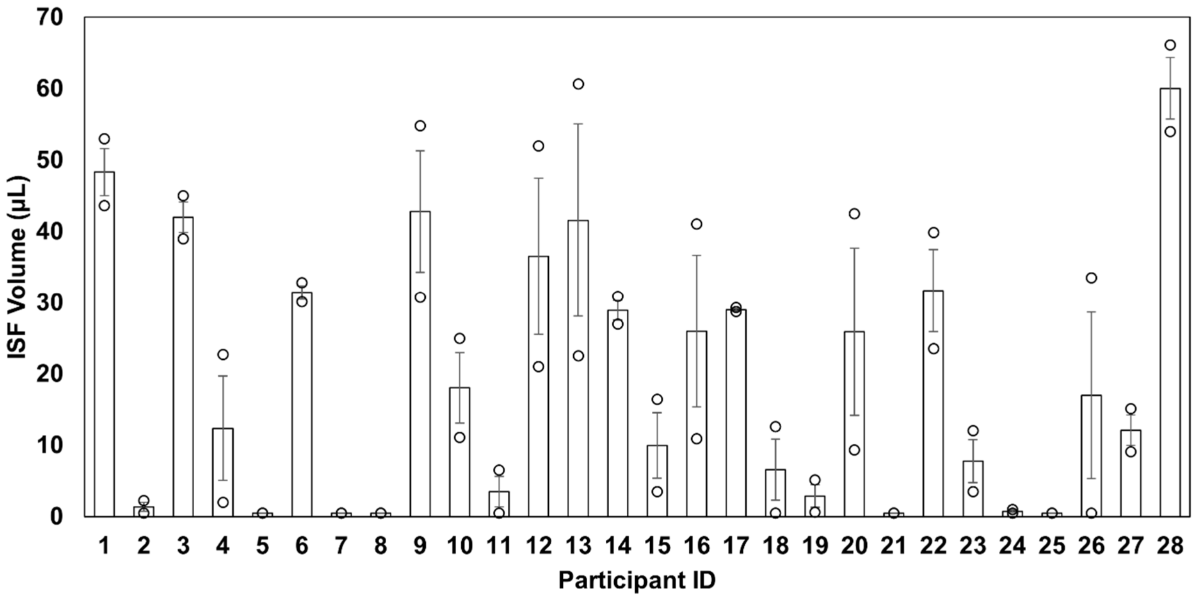

Figure S5. ISF collection volume from each participant. Each dot represents the volume of dermal ISF collected from one sample collection. Bars represent the average collection volume sampled from each participant from two independent sample collections.

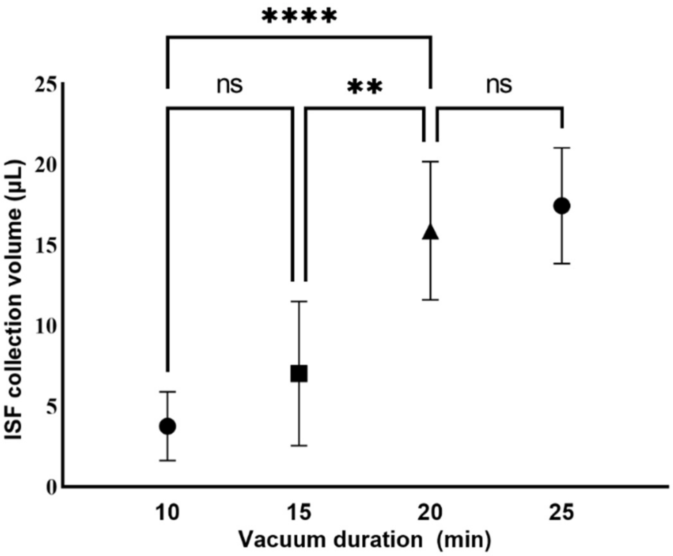

Figure S6. Influence of vacuum duration on ISF collection volume. ISF collection volume obtained using varying durations of applied vacuum. Experiments were performed using 20 × 20 MN arrays with needle lengths of 450 µm or 600 µm and three MN insertions per application site. Each data point represents the mean ± SD obtained from six independent sample collections (n=6). Significance was determined by one-way ANOVA with Tukey's post hoc (**p=0.0028, ****p<0.0001).

Cell Reports
Physical Science

(a) **PAIN SCALE**

0 — Pain free

1 — Very minor annoyance-occasional minor twinges

2 — Minor annoyance-occasional

3 — Annoying enough to be distracting

4 — Can be ignored if you are really involved in your work, but still distracting,

5 — Can't be ignored for more than 30 minutes.

6 — Can't be ignored for any length of time, but you can still go to work and participate in social activities.

7 — Make it difficult to concentrate, interferes with sleep, you can still function with effort

8 — Physical activity severely limited. You can read and converse with effort. Nausea and dizziness may occur.

9 — Unable to speak, crying out or moaning uncontrollable- pain makes you pass out

10 — Unconscious. Pain makes you pass out.

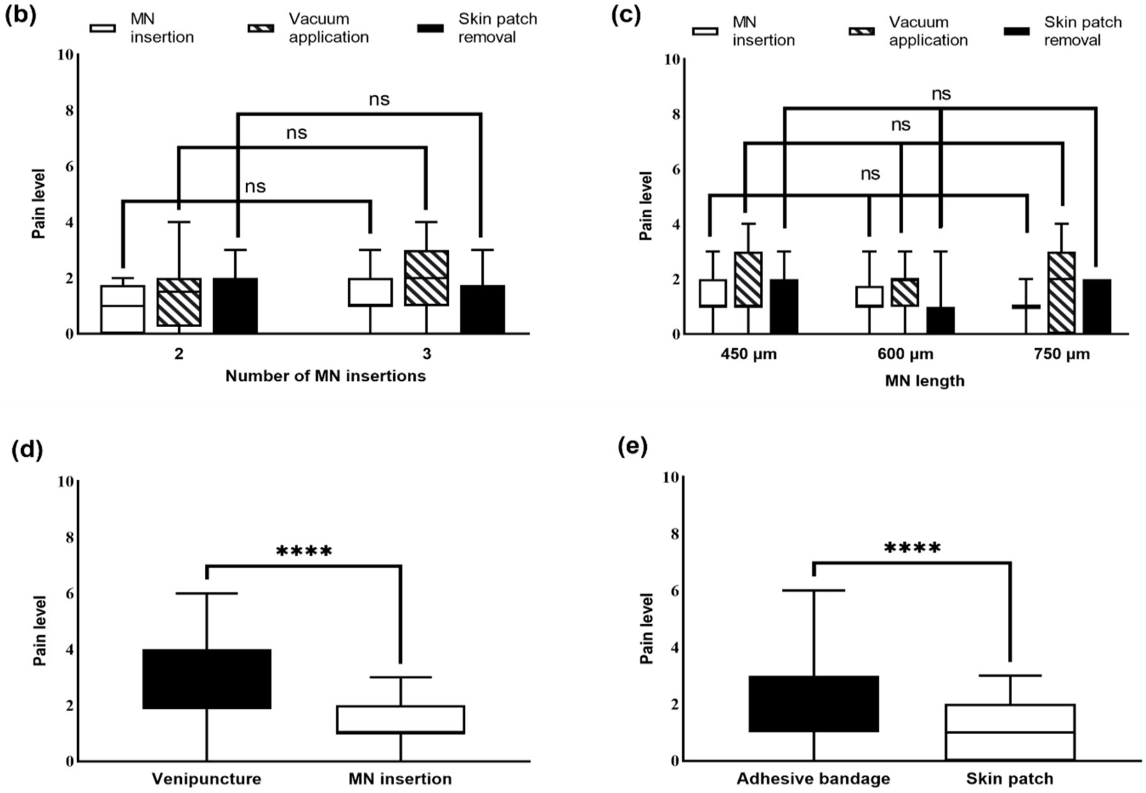

Figure S7. Self-reported pain levels associated with the ISF sampling procedure. (a) Pain level rating scale. (b) Participants' responses for pain level associated with different number of MN insertions per application site. Statistics acquired by Student's *t* test (ns=P>0.05). (c) Participants' responses for pain level associated with different MN lengths. Significance was determined by one-way ANOVA with Tukey's post hoc (ns=P>0.05). For comparison purposes, participants also rated their perceived pain levels during a standard venipuncture procedure and standard bandage removal. Participants' responses for comparing pain level between (d) MN insertion vs. venipuncture and (d) removal of the skin patch vs. removal of a standard adhesive bandage. Significance was determined with Student's *t* test (****p<0.0001). Each bar represents the mean ± min/max of responses from participants (n=26).

Cell Reports Physical Science

CellPress

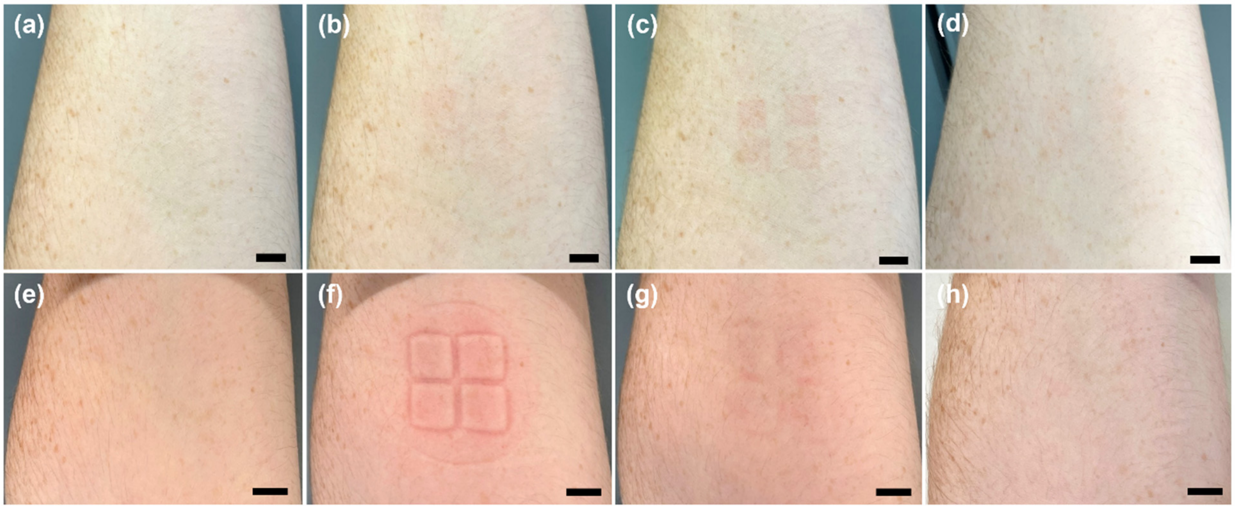

Figure S8. Effects of ISF sampling on the skin. Photographs of the sampling site from one participant (a) before MN insertion, (b) immediately after MN insertion, (c) 2 hr after MN insertion, and (d) 24 hr after MN insertion without vacuum application. Photographs of the sampling site from one participant (e) before ISF sampling, (f) immediately after ISF sampling, (g) 2 hr after ISF sampling, and (h) 24 hr after ISF sampling. Scale bars, 10 mm.

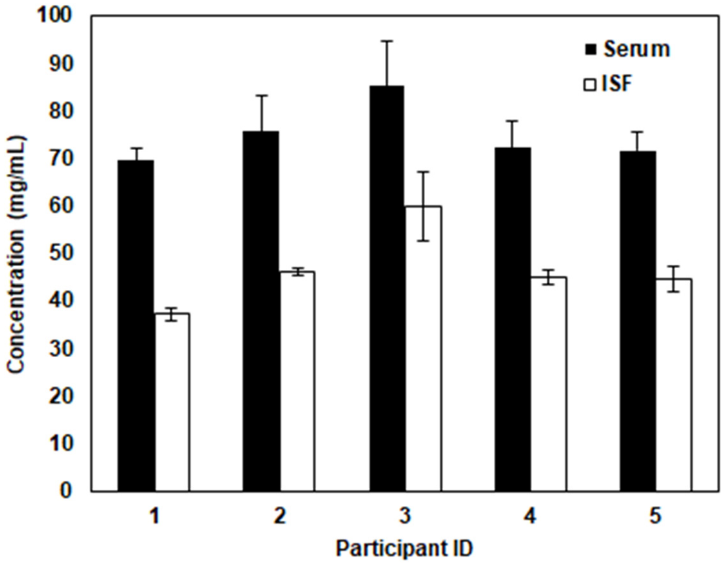

Figure S9. Absolution protein concentration in paired dermal ISF and blood serum samples from five volunteers. Each bar represents the mean ± SD of three measurements (n=5 participant triplicates).

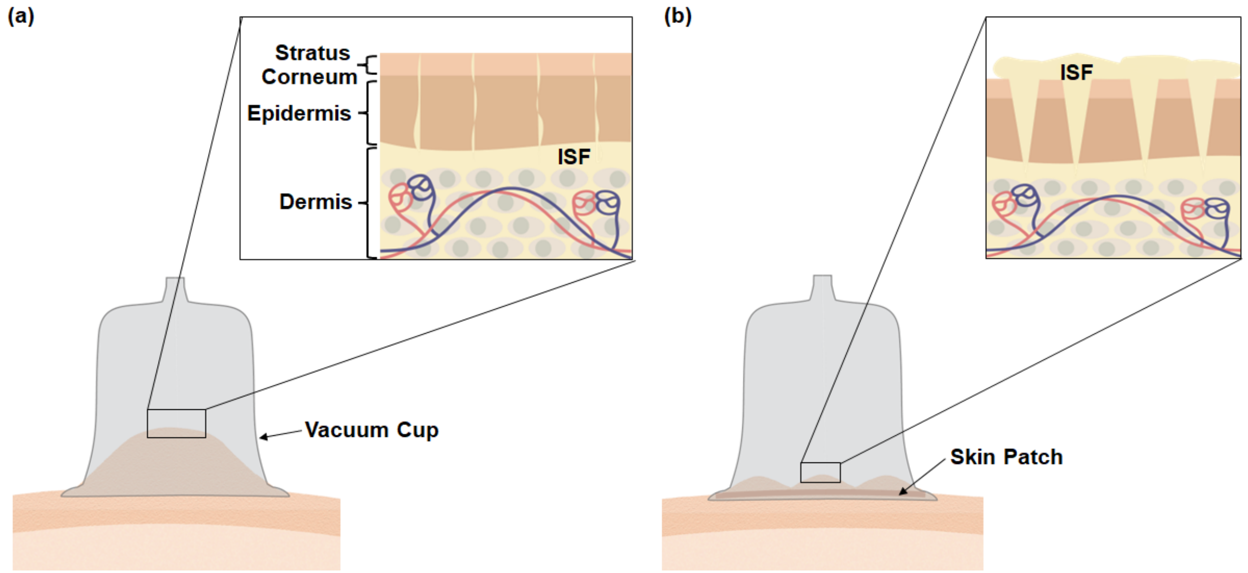

Figure S10. Schematic illustrations depicting ISF sampling with and without the skin patch. (a) Without the skin patch, the skin deforms excessively when suction is applied, causing the micropores to close. (b) With the skin patch, the skin is made taut when suction is applied, which induces the opening of the micropores, facilitating ISF extraction.

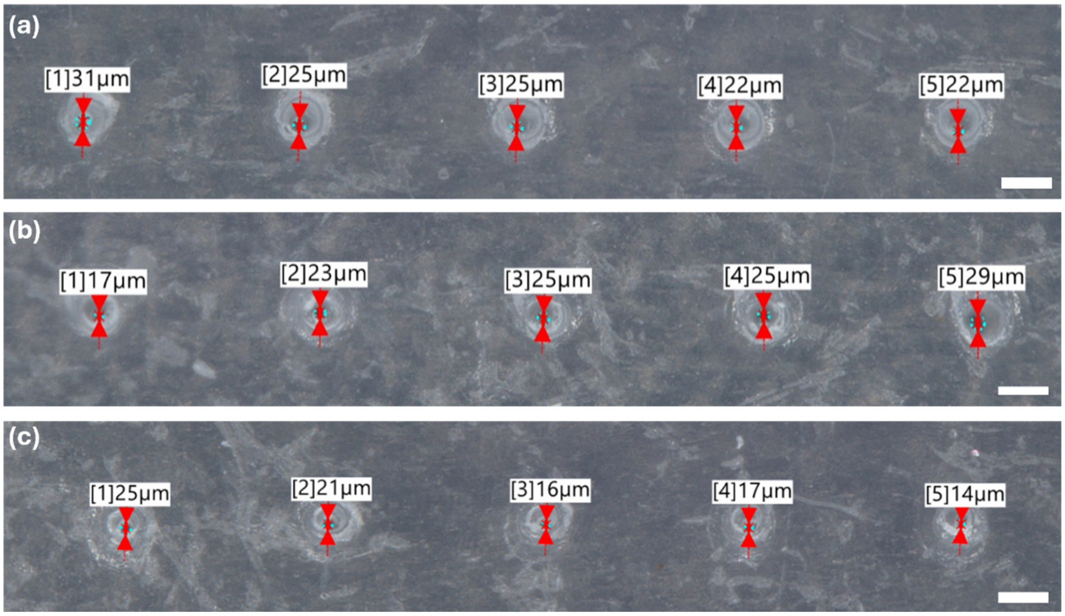

Figure S11. Micropore size as a function of MNs length. The insertion of MNs into a wax-based membrane model resulted in pore sizes of (a) 25 ± 3.67 μm for 450 μm-long MNs, (b) 23.8 ± 4.38 μm for 600 μm-long MNs, and (c) 18.6 ± 4.39 μm for 750 μm-long MNs. Each set of data represents the mean ± SD of 5 pores (n=5). Scale bars, 100 μm.

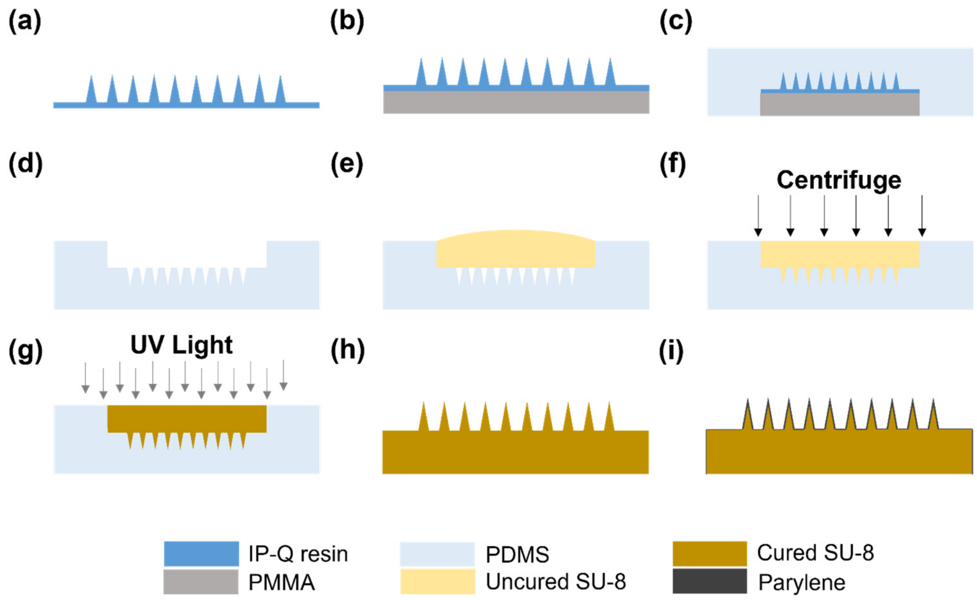

Figure S12. MN array fabrication process. (a) MN array master is fabricated using a NanoScribe 3D printer. (b) A 3 mm-thick PMMA substrate is attached to the backside of the MN array. (c)-(d) PDMS master mold is made via replica molding. (e) SU-8 is dropcasted on the PDMS mold. (f) Mold is centrifuged at 4,000 g for 15 min. (g) Exposure to 365 nm UV light for 3 min. (h) Polymerized SU-8 MN array is removed from PDMS mold. (i) MN array is coated in a layer of parylene.

Table S1. Demographics of the 28 participants enrolled in the ISF collection study.

**Age (years)**	
Mean ± SD	24.1 ± 3.8
Median	23.5
**Sex**	
Male	13
Female	15
**Ethnicity**	
Caucasian	7
African American	2
Hispanic	2
Asian	14
Multiple	3

Table S2. Demographics of the 5 participants enrolled in the ISF collection study whose samples were analyzed using LC-MS/MS.

Age (years)	
Mean ± SD	29.6 ± 5.1
Median	28
**Sex**	
Male	2
Female	3
**Ethnicity**	
Caucasian	1
Asian	3
Prefer not to answer	1

Table S3. Comparison of MN-based techniques for sampling ISF from human skin.

Type of MN	Equipment Needed for ISF Extraction	ISF Extraction Time	Average Collection Volume (μL)	Reference
Hydrogel	None	6 hr	-	[S1]
Hollow	None	5 min	1.1	[S2]
Hollow	None	15-20 min	< 1	[S3]
Hollow	Capillary tubes	1-2 hr	16	[S4, S5]
Solid	Electrical vacuum pump	20 min	2.3 ± 2.1	[S6]
Solid	Electrical vacuum pump	20 min	3.4 ± 3.2	[S7]
Solid	Vacuum cup and hand pump	20 min	20.8 ± 19.4	This work

Supplemental References

S1. Al-Kasasbeh, R., Brady, A.J., Courtenay, A.J., Larrañeta, E., McCrudden, M.T.C., O'Kane, D., Liggett, S., and Donnelly, R.F. (2020). Evaluation of the clinical impact of repeat application of hydrogel-forming microneedle array patches. Drug Deliv. Transl. Res. *10*, 690–705. 10.1007/s13346-020-00727-2.

S2. Ribet, F., Dobielewski, M., Böttcher, M., Beck, O., Stemme, G., and Roxhed, N. (2020). Minimally invasive and volume-metered extraction of interstitial fluid: bloodless point-of-care sampling for bioanalyte detection. Sens. Bio-Sensing Res.

S3. Mukerjee, E. V., Collins, S.D., Isseroff, R.R., and Smith, R.L. (2004). Microneedle array for transdermal biological fluid extraction and in situ analysis. Sensors Actuators, A Phys. *114*, 267–275. 10.1016/j.sna.2003.11.008.

S4. Tran, B.Q., Miller, P.R., Taylor, R.M., Boyd, G., Mach, P.M., Rosenzweig, C.N., Baca, J.T., Polsky, R., and Glaros, T. (2018). Proteomic Characterization of Dermal Interstitial Fluid Extracted Using a Novel Microneedle-Assisted Technique. J. Proteome Res. *17*, 479–485. 10.1021/acs.jproteome.7b00642.

S5. Miller, P.R., Taylor, R.M., Tran, B.Q., Boyd, G., Glaros, T., Chavez, V.H., Krishnakumar, R., Sinha, A., Poorey, K., Williams, K.P., et al. (2018). Extraction and biomolecular analysis of dermal interstitial fluid collected with hollow microneedles. Commun. Biol. *1*. 10.1038/s42003-018-0170-z.

S6. Samant, P.P., and Prausnitz, M.R. (2018). Mechanisms of sampling interstitial fluid from skin using a microneedle patch. Proc. Natl. Acad. Sci. U. S. A. *115*, 4583–4588. 10.1073/pnas.1716772115.

S7. Samant, P., Niedzwiecki, M. M., Raviele, N., Tran, V., Lapaix, J. M., Walker, D. I., Felner, E. I., Jones, D. P., Mil-ler, G. W. & Prausnitz, M.R. (2020). Sampling interstitial fluid from human skin using a microneedle patch. Sci. Transl. Med. *In Press*, 1–16.
