# Supplementary material for: Microneedle-based sampling of dermal interstitial fluid using a vacuum-assisted skin patch
Source: Cell Rep Phys Sci. 2024 Jun 19;5(6):101975. doi: 10.1016/j.xcrp.2024.101975 (PMC11211974; doi:10.1016/j.xcrp.2024.101975)
Supplement: Document S2. Article plus supplemental information [file mmc3.pdf]

Cell Reports
Physical Science

CellPress
OPEN ACCESS

Article

Microneedle-based sampling of dermal interstitial fluid using a vacuum-assisted skin patch

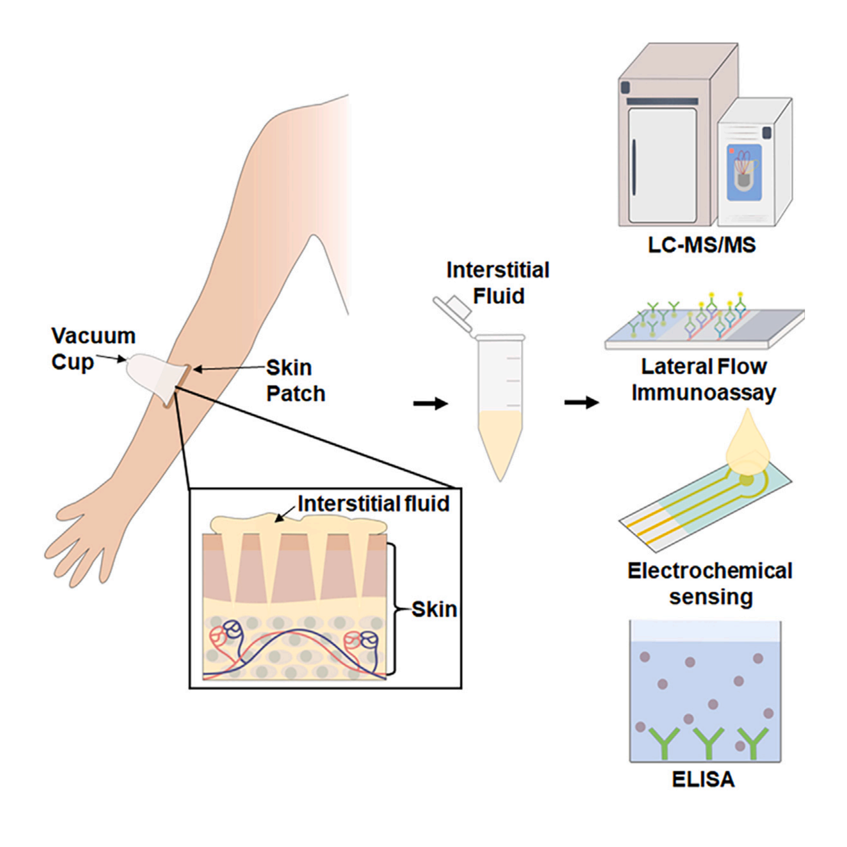

Jiang and Wilkirson et al. present a simple and minimally invasive technique for rapidly sampling interstitial fluid from human skin. Using this technique, interstitial fluid can be collected at ample quantities for biomolecular analysis using conventional techniques, such as liquid chromatography-mass spectrometry, western blot, enzyme-linked immunosorbent assays, and electrochemical sensing.

Xue Jiang, Elizabeth C. Wilkirson, Aaron O. Bailey, William K. Russell, Peter B. Lillehoj

lillehoj@rice.edu

Highlights

Minimally invasive technique for rapid, high-volume sampling of interstitial fluid

An average of 20.8 μL of human interstitial fluid is collected within 25 min

More than 600 medically relevant protein biomarkers are identified

SARS-CoV-2 neutralizing antibodies are detected from COVID-19 vaccinees

Jiang et al., Cell Reports Physical Science 5, 101975
June 19, 2024 © 2024 The Author(s). Published by Elsevier Inc.
https://doi.org/10.1016/j.xcrp.2024.101975

Check for updates

Cell Reports
Physical Science

CellPress
OPEN ACCESS

Article

Microneedle-based sampling of dermal interstitial fluid using a vacuum-assisted skin patch

Xue Jiang,[1,4] Elizabeth C. Wilkirson,[1,4] Aaron O. Bailey,[2] William K. Russell,[2] and Peter B. Lillehoj[1,3,5,*]

SUMMARY

Interstitial fluid (ISF) contains a wealth of biomolecules, yet it is underutilized for diagnostic testing due to a lack of rapid and simple techniques for collecting abundant amounts of fluid. Here, we report a simple and minimally invasive technique for rapidly sampling larger quantities of ISF from human skin. A microneedle array is used to generate micropores in skin from which ISF is extracted using a vacuum-assisted skin patch. Using this technique, an average of 20.8 μL of dermal ISF is collected in 25 min, which is an ~6-fold improvement over existing sampling methods. Proteomic analysis of collected ISF reveals that it has nearly identical protein composition as blood, and >600 medically relevant biomarkers are identified. Toward this end, we demonstrate the detection of SARS-CoV-2 neutralizing antibodies in ISF collected from COVID-19 vaccinees using two commercial immunoassays, showcasing the utility of this technique for diagnostic testing.

INTRODUCTION

The detection and quantification of biomolecules in bodily fluids plays an important role in medicine. Currently, the diagnosis and monitoring of many diseases relies on the analysis of blood for the presence of biomolecular markers. While blood sampling is a routine medical procedure, it poses risks of infection[1] and can lead to complications in infants and individuals with blood clotting disorders.[2] Furthermore, the pain associated with blood sampling can deter individuals with needle or blood phobias from getting tested.[1] Urine and saliva are less invasive and easier to collect; however, these fluids contain only subsets of the biomarkers found in blood,[3] typically at significantly lower concentrations,[4] hindering their use for many diagnostic applications.[5]

Interstitial fluid (ISF) is a fluid that surrounds cells and tissues and accounts for 15%–25% of the total human body weight.[6] ISF is most abundantly found in the lower viable epidermis and the upper dermis,[7,8] which is composed of ISF by up to 70% by volume.[6] Prior studies have shown that ISF collected from skin (i.e., dermal ISF) contains many of the same biomolecules, including metabolites, proteins, and nucleic acids, as blood.[8–13] For example, glucose has been detected in dermal ISF, and its concentration was shown to be highly correlated with concentrations in blood plasma and serum.[8,14] Additionally, the pharmacodynamics of glucose in children and young adults and the pharmacokinetics of caffeine in healthy adults were shown to be similar in human ISF and plasma.[15,16] In addition to biomarkers associated with systemic physiology, dermal ISF contains local biomarkers associated with skin and tissue physiology that are not found in blood,[15] making it potentially useful for the diagnosis of skin conditions and disorders.

[1]Department of Mechanical Engineering, Rice University, Houston 77005, TX, USA
[2]Mass Spectrometry Facility, University of Texas Medical Branch, Galveston 77550, TX, USA
[3]Department of Bioengineering, Rice University, Houston 77030, TX, USA
[4]These authors contributed equally
[5]Lead contact
*Correspondence: lillehoj@rice.edu
https://doi.org/10.1016/j.xcrp.2024.101975

This is an open access article under the CC BY license (http://creativecommons.org/licenses/by/4.0/).

While dermal ISF is a promising source of molecular biomarkers, its use for diagnostic testing is hampered by the lack of rapid and simple techniques for collecting abundant amounts of fluid.[17,18] Various methods for extracting ISF from skin, including microdialysis,[19] open-flow microperfusion,[20] laser microporation,[21] or reverse iontophoresis,[22] have been reported; however, they are invasive, time consuming (~1 h), require specialized equipment, and need to be performed by trained medical professionals.[23] One commonly used approach for collecting ISF involves the creation of suction blisters to draw fluid to the skin, which is subsequently collected using a hypodermic needle and syringe.[10,11,15] While effective, this method requires at least 1 h for blistering to occur and can cause prolonged skin erythema and dehydration at the sampling site.[10] Furthermore, ISF obtained via suction blister contains biomarkers associated with tissue injury, making it less representative of physiologic ISF.[11]

An alternative strategy for sampling ISF uses microneedles (MNs) to penetrate the skin, providing access to ISF in the upper dermis. Compared to hypodermic needles, MNs avoid the nerves and vascular structures located in the deeper layers of the dermis (starting at ~1,500 μm below the skin surface), thereby significantly minimizing their associated pain and risks of infection.[24] MNs have been extensively studied for minimally invasive transdermal drug and vaccine delivery, but less research has been reported on using them to extract ISF in humans.[25] Kasasbeh et al. reported the use of hydrogel-based MNs to extract ISF from human skin; however, this approach required 6 h of MN application and involved time-consuming and tedious procedures to extract fluid from the MN array.[26] Mukerjee et al. demonstrated the extraction of ISF from human skin using a microfluidic device consisting of a hollow MN array connected to a series of microchannels.[27] For proof of concept, this device was applied to the author's earlobe for 15–20 min, resulting in the extraction of a small droplet (~50 μm in diameter) of ISF. In another study, a hypodermic-based MN device was used to extract 1.1 μL of ISF in 5 min from the forearm.[12] Studies by Samant et al. have demonstrated the collection of dermal ISF from human skin using solid metal MNs, which yielded volumes between ~1 and 6 μL.[8,15] While these techniques are capable of extracting ISF from human skin, the collected volumes are too low for biomolecular analysis using conventional diagnostic assays, such as enzyme-linked immunosorbent assay (ELISA), western blot, or lateral flow immunochromatographic assay (LFIA). Miller et al. reported a method for sampling ISF from human skin using hollow microneedles that could extract up to 16 μL of ISF; however, this approach required several hours and continual re-application (every 30 min) of the MNs.[13]

Here, we report a simple and minimally invasive technique for rapidly sampling larger quantities of ISF from human skin. In this approach, micropores are generated in the skin using a high-density MN array, followed by the attachment of a rigid skin patch and application of mild vacuum pressure using a portable hand pump. MN arrays of varying sizes and needle lengths were fabricated and characterized to investigate their mechanical strength, skin penetration effectiveness, and ISF collection performance. Parameters associated with the sample collection process, including the number of MN insertions and the duration of vacuum application, were studied to optimize the ISF sampling efficiency. Pain levels and skin tolerability were also investigated to assess the safety and acceptability of this technique. Dermal ISF and fingerstick blood collected from human volunteers were analyzed using nanoflow liquid chromatography-tandem mass spectrometry (LC-MS/MS) to compare their protein composition and evaluate the diagnostic utility of ISF obtained using this method. Dermal ISF collected from COVID-19 vaccinees was also analyzed for

CellPress
OPEN ACCESS

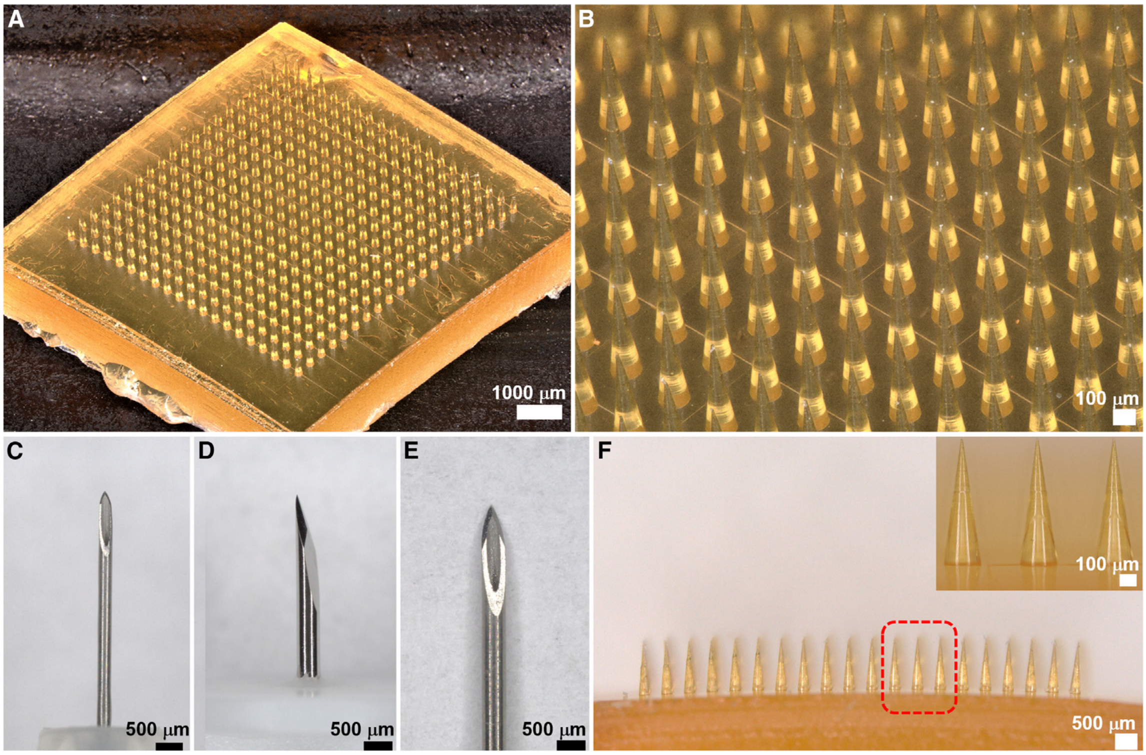

Figure 1. Images of the MN array and conventional needles

(A) Optical micrograph of the 20 × 20 MN array at 20× magnification. Scale bar, 1,000 μm.

(B) Close-up view of the 20 × 20 MN array at 80× magnification. Scale bar, 100 μm.

(C–E) Optical micrographs of a (C) 32G pen needle, (D) 28G lancet, and (E) 27G hypodermic needle tip at 40× magnification for size comparison to the MNs. Scale bars, 500 μm.

(F) Side view of the 20 × 20 MN array at 40× magnification. Scale bar, 500 μm. Inset shows a close-up view of the MNs at 200× magnification. Scale bar, 100 μm.

SARS-CoV-2 neutralizing antibodies using two commercially available immunoassays to demonstrate the utility of this approach for ISF-based diagnostic testing.

RESULTS

Design and characterization of the MN array

The MN arrays are composed of solid, conical MNs made from polymerized SU-8 photoresist (Figures 1A and 1B). While polymerized SU-8 is a biocompatible material with low cytotoxicity and minimal reaction in tissue,[28] MNs were coated with 1.5 μm of parylene to further enhance their biocompatibility.[29] MNs were designed to safely penetrate human skin multiple times to create thousands of micropores while maintaining a compact profile to minimize discomfort. MN arrays with three different needle heights (450, 600, and 750 μm) were fabricated to determine the optimal length for extracting the greatest amount of ISF with the least amount of pain. A base diameter of 200 μm was used for all the MNs. The overall size of the MNs is considerably smaller than conventional needles commonly used for blood sampling and intravenous therapy, such as pen needles, lancet needles, and hypodermic needles (Figures 1C–1E), which significantly minimizes the pain and skin reactions associated with their insertion into human skin. MNs were configured in a two-dimensional array (10 × 10 or 20 × 20) to multiply the number of micropores generated per insertion, with a needle-to-needle spacing of 400 μm for each size array (Figure 1F). The overall sizes of the 10 × 10 and 20 × 20 arrays were 7.5 × 7.5 mm and 10 × 10 mm, respectively.

The mechanical strength of the MN arrays was characterized to assess their ability to safely penetrate human skin. Force-displacement curves were generated of 10 × 10 and 20 × 20 MN arrays with needle lengths of 450, 600, and 750 μm subjected to mechanical compression (Figure S1). The 10 × 10 and 20 × 20 MN arrays did not exhibit any signs of deformation when compressed up to 50 N, which is at least 1.5-fold larger than the force required to penetrate human skin (0.08 N per MN).[30] Subjecting the MN arrays to compression >50 N caused the tips of the MNs to undergo plastic deformation (Figure S2); however, none of the MNs exhibited signs of failure (i.e., fracture). These results indicate that the MNs can penetrate human skin and will not break during skin insertion, thereby eliminating potential complications associated with MN failure.

We assessed the capability of the MNs to generate micropores in skin by applying the MN arrays to porcine skin, which was used as an anatomically and biochemically similar model as human skin.[31] Prior to skin insertion, MNs were coated with blue ink for improved visualization. Distinct micropores were generated by each MN, which were confined to the needle penetration sites with no impact to the surrounding tissue (Figures 2A and 2B). Histological analysis was also performed to evaluate the effects of microneedle penetration in skin tissue. Each MN insertion site was characterized by a conical micropore that pierces through the epidermis (Figures 2C–2E). The formation of these cavities provides access to dermal ISF in the upper dermis while avoiding the dense collection of nerves and vascular structures located in the lower dermis layer. MN integrity was evaluated by applying the MN array to skin multiple times. Skin samples were punctured 36 times using the 10 × 10 MN arrays and 12 times using the 20 × 20 MN arrays, which are the maximum number of times that the MNs are inserted into the skin to generate micropores for ISF extraction. Optical micrographs of the MN array following repeated skin insertion revealed that the MNs exhibited no discernable deformation or damage (Figure S3).

Vacuum-assisted sampling of ISF from human skin using the skin patch

ISF was collected from 28 adults at Rice University, whose demographics are listed in Table S1. The ISF sampling procedure is shown in Figures 3A–3D. A double-sided skin-friendly adhesive sticker was first adhered to the anterior forearm, which was selected as the ISF sampling site due to its ease of access and lack of excess body hair. The sticker contains rectangular cutouts, which serve as guides for the MN insertion sites. For the 10 × 10 MN array, the cutouts are arranged in three columns, resulting in 12 distinct MN insertion sites (Figure S4A). For the 20 × 20 MN array, the cutouts are arranged as a 2 × 2 matrix, resulting in four distinct MN insertion sites (Figure S4B). The MN array was applied to the skin two or three times at each insertion site using a spring-loaded applicator (Figure 3A), which generated thousands of micropores in the skin in a rapid and repeatable manner. A rigid plastic plate (containing identical cutouts as the sticker) was adhered to the sticker, followed by the attachment of a vacuum cup. Vacuum pressure (−44 kPa) was generated inside the cup using a hand pump (Figure 3B), resulting in pressure-driven convection of ISF through the micropores. The skin patch kept the skin taunt when vacuum pressure was applied, which induced the opening of the micropores for increased ISF extraction. The extracted ISF was pooled on the skin forming small droplets, which were confined within the rectangular wells of the patch (Figure 3C). After 20 min, the vacuum cup was removed, and the ISF was collected using capillary tubes (Figure 3D).

ISF collected using this technique was clear to light yellowish in color and generally more viscous than sweat (Figures 3E–3H), which is consistent with prior observations of ISF extracted from human skin.[32] Precautions were taken during the sampling

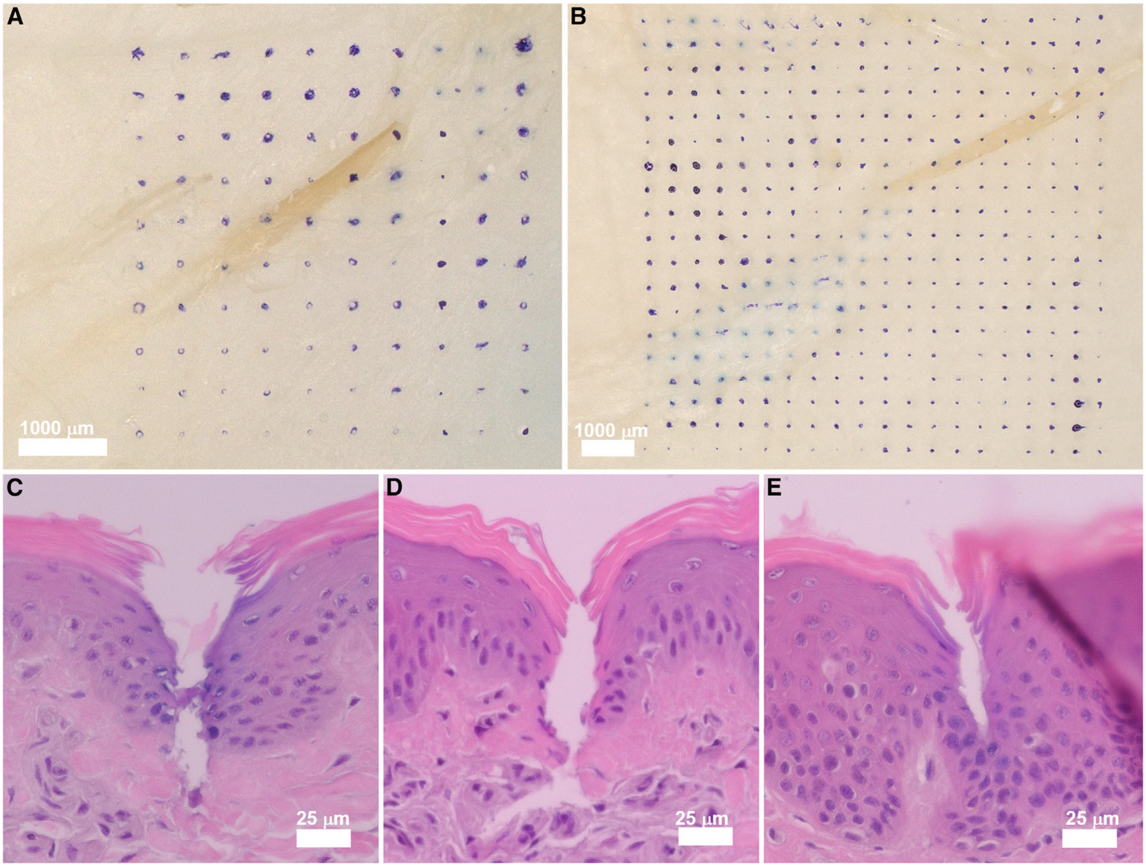

Figure 2. Skin penetration performance of the MN array

(A and B) Distinct micropores generated in porcine skin using the (A) 10 × 10 MN array and (B) 20 × 20 MN array at 20× and 30× magnification, respectively. Scale bars, 1,000 μm. Prior to skin insertion, MNs were coated with blue ink for improved visualization.

(C–E) H&E-stained section of porcine skin penetrated by MNs with length of (C) 450 μm, (D) 600 μm, and (E) 750 μm at 1,000× magnification. Scale bars, 25 μm.

procedure to ensure that the ISF sample was not contaminated with sweat. Sample collection was performed in a temperature- (68°F–72°F) and humidity- (~40–50% RH) controlled environment to minimize perspiration. Prior to sample collection, the participant's skin was cleaned with an alcohol prep pad and thoroughly dried, further reducing the likelihood of sample contamination with sweat or environmental residues. We investigated whether the ISF sampling procedure could cause the secretion of sweat by adhering the skin patch to the forearm of a volunteer and applying suction for 20 min using the vacuum cup (without MN insertion). No fluid was observed on the skin, confirming that the ISF sample was not contaminated with sweat. Microscopic inspection of collected ISF revealed the absence of red blood cells, which are markers for whole blood contamination,[33] thus confirming that the ISF samples were not contaminated with blood.

The average amount of ISF collected from all 28 participants was 20.8 ± 19.4 μL (mean ± standard deviation [SD]), where up to 66.1 μL was collected from one participant. The amount of ISF collected from each participant is presented in Figure S5. Variability in the amount of dermal ISF collected from different individuals using the same MN parameters and sample collection procedure was observed. We attribute this variability to differences in the participants' skin, such as topology, thickness, elasticity, and hydration, which can affect the MN penetration depth

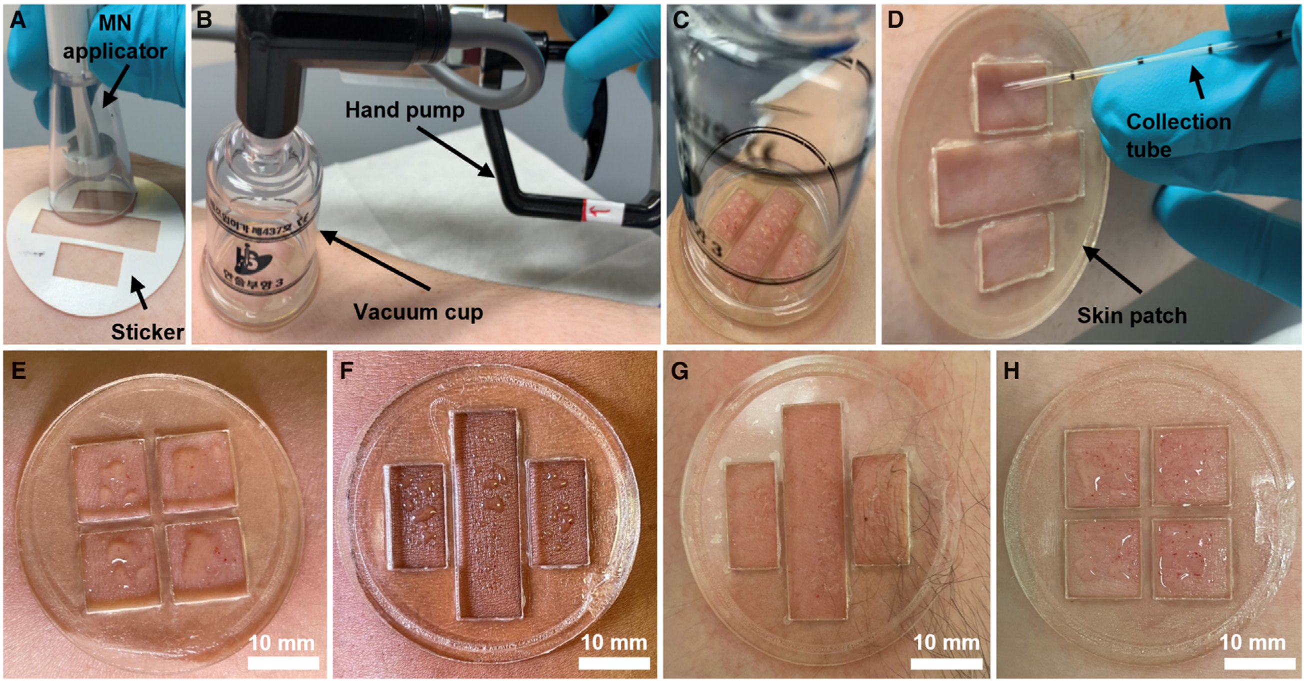

Figure 3. ISF sampling procedure and representative images of extracted ISF

(A) The skin patch sticker is adhered to the anterior forearm, followed by MN insertion using the MN applicator.

(B) The rigid plate is attached to the sticker, followed by the attachment of a vacuum cup. Vacuum pressure is generated in the cup using a hand pump.

(C) Vacuum pressure is maintained for 20 min for ISF extraction.

(D) The vacuum cup is removed, and the extracted ISF is collected using capillary tubes.

(E–H) Extracted ISF on the skin of four volunteers. Scale bars, 10 mm.

and micropore size. Intrasubject and intersubject variability in the sample collection volume was also reported by Samant et al. using an MN- and vacuum-assisted technique for extracting ISF from skin.[15] Furthermore, prior studies have shown variability in the collection of other bodily fluids, including blood,[34] sweat,[35] and saliva,[36] among different individuals.

We investigated the influence of several MN parameters, including the array size (10 × 10 and 20 × 20), needle length (450, 600, and 750 μm), and the number of MN insertions (two or three) per application site, on the amount of ISF that could be collected from participants (Figures 4A and 4B). We observed that the MN length did not have a significant effect on the ISF collection volume when applied two times per application site. However, the 450-μm-long MNs resulted in a significantly larger amount of ISF than the 600- and 750-μm-long MNs when applied three times per application site. Additionally, in a 20 × 20 array format, the 450-μm-long needles resulted in the collection of significantly more ISF than the 750-μm-long MNs. Based on these collective results, using a 20 × 20 MN array with a needle length of 450 μm and three MN insertions per application site resulted in the greatest amount of collected ISF.

The influence of the vacuum duration on the ISF collection volume was also studied by varying the amount of time that suction was applied to the skin. We observed a positive correlation between the vacuum duration and the ISF collection volume where longer durations of applied vacuum resulted in the extraction of larger amounts of ISF (Figure S6). There was a significant increase in the amount of ISF collected from participants by applying suction for 20 min compared with 10 or

CellPress
OPEN ACCESS

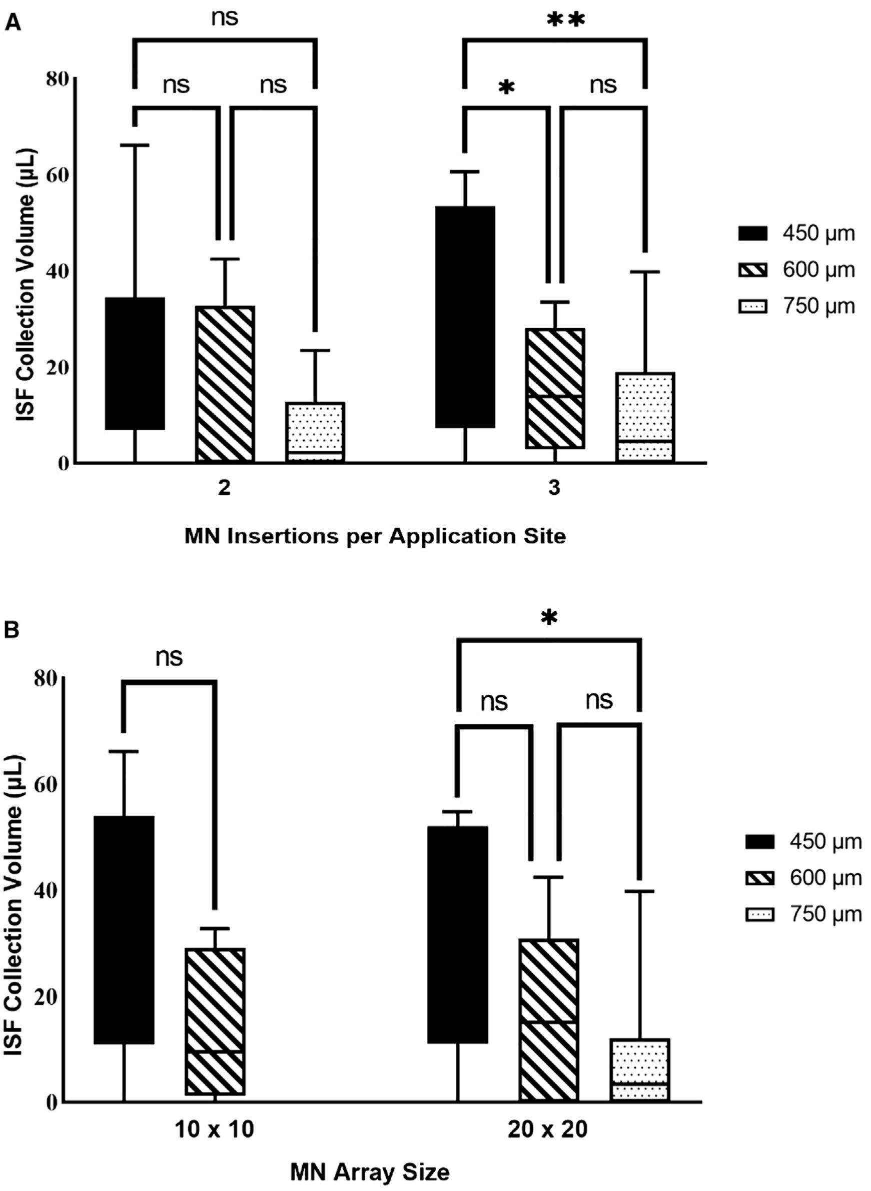

Figure 4. Influence of MN parameters on ISF collection volume

(A) Volume of ISF collected using needle lengths of 450 µm, 600 µm, or 750 µm with two or three MN insertions per application site. Significance for two insertions was determined using one-way ANOVA with Tukey's post hoc (ns = $p > 0.05$), and significance for three insertions was determined by two-way ANOVA with Tukey's post hoc (ns = $p > 0.05$, *$p = 0.0323$, **$p = 0.0303$).

(B) Volume of ISF collected using needle lengths of 450 µm, 600 µm, or 750 µm configured in 10 × 10 or 20 × 20 arrays. Significance for the 10 × 10 array was determined using Student's t test (ns = $p > 0.05$), and significance for the 20 × 20 array was determined by one-way ANOVA with Tukey's post hoc (ns = $p > 0.05$, *$p = 0.0113$).

15 min. However, there was not a significant increase in the ISF volume when suction was applied for >20 min, which is consistent with prior studies showing that ISF extracted from the skin plateaus after ~20 min.[32] Furthermore, minor complications, like erythema, edema, and ecchymosis, can occur to the skin when suction is applied for >20 min.[37] Therefore, 20 min was selected as the optimal duration for vacuum application following MN insertion.

Participants completed a survey to rate the pain level (based on the Mankoski Pain Scale,[38] which ranges from 0 to 10, with 0 being painless and 10 being unbearable; Figure S7A) associated with the MN insertion, vacuum application, and removal of the skin patch (Figures S7B–S7E). The pain level reported for the entire sample collection procedure by all participants was 1.27 $\pm$ 1.03 (mean $\pm$ SD), where 33% of the participants rated the pain level as <1 (pain free). We also investigated whether the ISF sampling procedure caused any adverse effects to the skin. MN insertion resulted in slight skin redness at the MN application sites, which resolved within 24 h (Figures S8A–S8D). More pronounced skin redness, mild swelling, and slight tenderness localized within the skin patch were observed as a result of suction being applied to the skin (Figures S8E–S8H); however, these reactions are common and benign effects associated with cupping/vacuum therapy.[39] Overall, the collection of ISF using this technique was well tolerated with minor adverse effects that completely resolved within 1 day.

Proteomic analysis of dermal ISF

Dermal ISF and blood were sampled from five volunteers (demographics listed in Table S2) and analyzed for protein composition using LC-MS/MS. We initially analyzed both fluids and found that they both contained a high level of abundant proteins, such as albumin and immunoglobulins. Therefore, the abundant proteins were removed from the fluids using a commercial protein depletion kit and reanalyzed. This analysis resulted in the identification of 2,195 distinct proteins, where 91.6% were common between both fluids, 4.7% were unique to blood serum, and 3.7% were unique to ISF (Figure 5A). To determine the differential abundance of proteins in each fluid, protein identifications were filtered for statistical significance and relative fold change, as shown in the volcano plot (Figure 5B). The proteomic results were further analyzed using two online biomarker databases (OncoMX and BIONDA) to identify medically relevant biomarkers. From these databases, 610 proteins detected in both ISF and serum with similar abundance ratios (within a $\log_2$ value of 0.50–2.0) are associated with diseases (Data S1). Of these proteins identified, 98 are classified in the NCI Early Detection Research Network (EDRN) biomarker database, and 5 are approved biomarkers by the US Food and Drug Administration (FDA), with 3 of these being both EDRN and FDA biomarkers. The abundance levels of proteins in ISF and serum determined by LC-MS/MS were based on relative measurements. Therefore, the absolute protein concentrations in the five paired ISF and serum samples were measured using a Bradford protein assay. From this analysis, the absolute concentrations of proteins in ISF and serum were 46.50 $\pm$ 8.25 mg mL^{-1} and 74.90 $\pm$ 6.23 mg mL^{-1}, respectively (Figure S9), which is consistent with prior studies analyzing the total protein content in these fluids.[40–43]

Detection of SARS-CoV-2 neutralizing antibodies in dermal ISF

We analyzed dermal ISF samples from COVID-19 vaccinees for the presence of SARS-CoV-2 neutralizing antibodies using two commercial SARS-CoV-2 neutralization antibody tests. Two paired dermal ISF and blood samples were first tested using an LFIA test. Dark test and control lines were generated with both samples for each participant, indicating the presence of SARS-CoV-2 neutralizing antibodies (Figure 5C). Dermal ISF from 15 COVID-19 vaccinees was also analyzed using an ELISA-based SARS-CoV-2 surrogate virus neutralization test kit to quantify the concentration of the SARS-CoV-2 neutralizing antibody in the samples. SARS-CoV-2 neutralization antibody was detected in dermal ISF from all vaccinees at concentrations ranging from 81 to 618 ng mL^{-1} (Figure 5D). These collective results provide evidence that dermal ISF is a source for biomarkers associated with vaccination

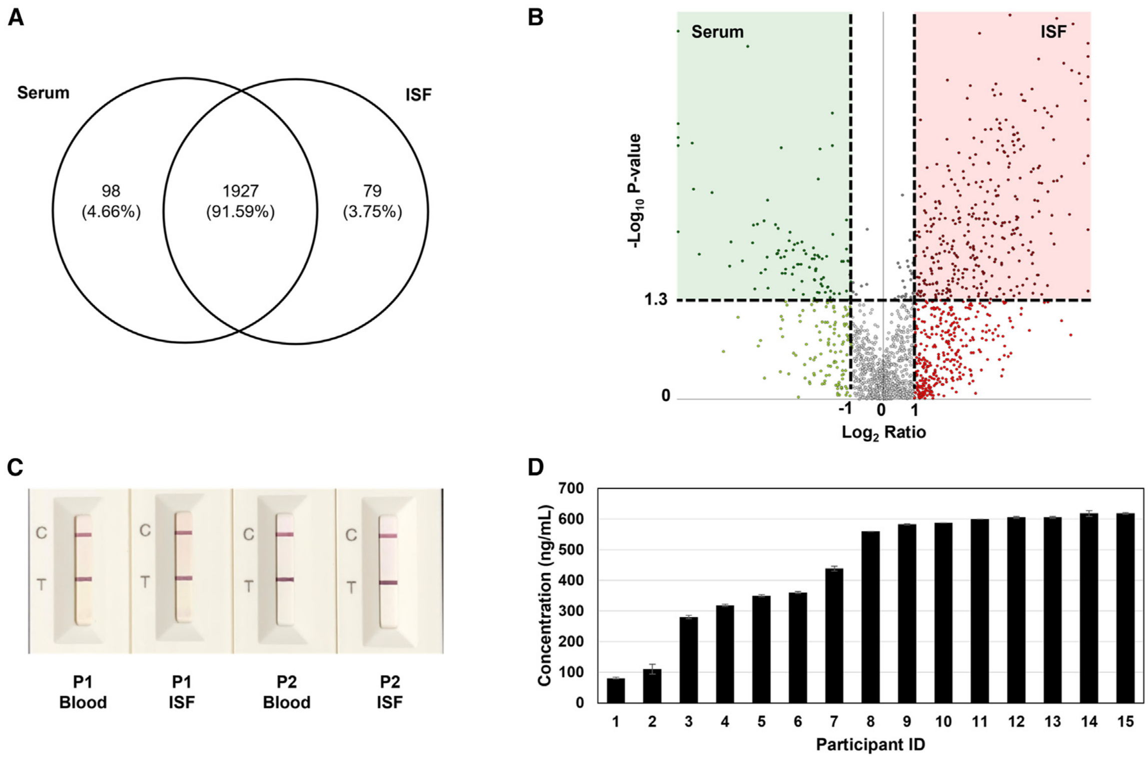

Figure 5. Proteomic analysis of dermal ISF

(A) Venn diagram showing the overlap in proteins identified in dermal ISF and blood serum samples obtained from five volunteers analyzed using LC-MS/MS.

(B) Volcano plot of all proteins identified in dermal ISF vs. blood serum samples. Statistical significance and differential abundance were determined using a minimum $-\text{Log}_{10}\ p$ value of 0.05 and a Log_2-fold change of 1, respectively. Points above the horizontal dashed line represent proteins with statistically significant identifications (p value > 0.05). Points to the left of the leftmost vertical dashed line denote abundance ratios of ISF/blood serum <0.5, while points to the right of the rightmost dashed line denote abundance ratios of ISF/blood serum >2. Points located in the red shaded region denote proteins that are upregulated in ISF, while points located in the green shaded region denote proteins upregulated in blood serum.

(C) LFIA test results of paired dermal ISF and blood serum samples obtained from two COVID-19 vaccinees (P1, P2) for the detection of SARS-CoV-2 neutralizing antibodies.

(D) Concentration of SARS-CoV-2 neutralizing antibodies in dermal ISF collected from 15 COVID-19 vaccinees (n = 15) measured using ELISA. Each bar represents the mean $\pm$ SD of two measurements.

status and further suggests that other molecular biomarkers associated with infection and disease status are presented in ISF.

DISCUSSION

Progress in the use of dermal ISF as a diagnostic fluid has been hampered by the lack of simple, rapid, and minimally invasive sampling methods capable of extracting larger quantities of fluid.[17] A major limitation of existing MN-based ISF sampling techniques is that the collected fluid volumes are too low for biomolecular analysis using commercially available diagnostic immunoassays (e.g., ELISA, western blot, and LFIA), which require at least 10–20 μL of fluid. Here, we present the development of a rapid (25 min), simple, and minimally invasive technique for sampling ample quantities of ISF from human skin, which was achieved by implementing several unique strategies. Existing MN-based ISF sampling methods employ MN arrays consisting of a few MNs, resulting in a small number of micropores generated in the skin, even with repeated MN insertion. In our approach, a high-density MN array was applied to the skin three times (MN insertions were not intentionally aligned), resulting in the generation of thousands of micropores from which ISF could be

extracted. More importantly, we hypothesize that the low sample volumes generated from the MN- and vacuum-assisted ISF sampling methods reported in prior studies are due to the high elasticity of human skin, which can deform excessively when vacuum pressure is applied,[44] causing the micropores to close (Figure S10A). Studies demonstrating the extraction of larger quantities (>6 μL)[45] of fluid through MN-generated micropores in excised animal skin or artificial skin models[46,47] utilized high vacuum pressures to induce the opening of the micropores. Applying such strong suction to human skin would be painful and cause skin injury. To overcome this challenge, we developed a rigid patch that is adhered to the skin, which serves two important functions. First, the patch creates an air-tight seal between the skin and vacuum cup, enabling vacuum pressure to be maintained throughout the ISF extraction process. Second, the patch keeps the skin taut when suction is applied, which induces the opening of the micropores, enabling pressure-driven convection of ISF through the micropores (Figure S10B). To validate the effectiveness of the skin patch for enhancing ISF extraction in human skin, the ISF sampling procedure was performed on volunteers without the patch. Upon applying suction to the vacuum cup, the skin deformed significantly (compared to when the patch was used), and no fluid was observed on the skin after 20 min. The mechanism for enhanced ISF extraction using the skin patch could be applied to other ISF sampling methods that have been reported in literature, which could lead to further improvements in their performance. Furthermore, we envision that this ISF sampling technique can be adapted onto a wearable biosensing platform, enabling *in situ* measurements of analytes in dermal ISF for point-of-care diagnostic testing.

We analyzed the amount of ISF extracted using differently sized MNs and found that the 450-μm-long needles yielded the largest volume of dermal ISF compared with the 600- and 750-μm-long needles. We attribute this to the 450-μm-long MNs creating larger diameter micropores compared to the 600- and 750-μm-long needles, which allows for more ISF to flow through the micropores. A base diameter of 200 μm was used for all the MNs; therefore, longer MNs have a more slender profile than shorter MNs, thereby creating smaller micropores in the skin. This was confirmed by measuring the pore size generated by MNs with the three different lengths when penetrated into a wax-based membrane model (Figure S11).[48] We observed intrasubject and intersubject variability in the amount of dermal ISF that could be collected using this sampling technique. Further studies will be needed involving the collection of dermal ISF from larger populations of individuals with a broad range of ages, ethnicities, and body mass indexes, which can lead to a deeper understanding of the mechanics of vacuum-assisted ISF extraction from human skin. Additional studies to further optimize the MN parameters and sample collection procedure from these populations could enhance the reliability (i.e., reduce variability) of ISF collection.

Proteomic analysis of dermal ISF and blood collected from five volunteers resulted in the identification of 2,195 distinct proteins with the majority of these appearing in both fluids. Of those found, 610 proteins detected in both ISF and serum with similar abundance ratios are recognized as medically relevant biomarkers according to the BIONDA and OncoMX databases. These biomarkers include ones for various types of cancers, neurodevelopmental disorders, inflammatory diseases, genetic disorders, and more. These data indicate that dermal ISF may be a source for many of the same biomarkers associated with illness, infection, and vaccination status that are present in blood. While we observed a significant overlap in protein composition in both fluids, there was also a small (~3.8%) subset of proteins that were only detected in dermal ISF, indicating that ISF could provide unique diagnostic and health

information that cannot be obtained from blood. Among these are proteins associated with inflammation (i.e., interleukin-37), physiological responses such as electrolyte secretion (i.e., calcium-activated chloride channel regulator 4), and cancers such as cutaneous T cell lymphoma (i.e., melanoma inhibitory activity protein 2). To further showcase the utility of this sampling technique for diagnostic testing, dermal ISF was collected from COVID-19 vaccinees and analyzed for SARS-CoV-2 neutralizing antibodies using two commercially available immunoassays. Using the LFIA-based test, SARS-CoV-2 neutralizing antibodies could be detected in a rapid (~15 min) and simple manner, while SARS-CoV-2 antibody levels could be quantified in the ISF samples using the ELISA-based test.

The sampling technique reported in this work represents a notable improvement over existing MN-based ISF sampling methods in the ability to rapidly extract larger ISF volumes in a minimally invasive manner without the use of specialized equipment. A comparison of this technique with other MN-based techniques for sampling ISF from human skin is presented in Table S3. In addition to its enhanced effectiveness in sampling dermal ISF, this technique was well tolerated by all participants with only minor adverse effects that completely resolved within 1 day. Furthermore, participants rated the sampling technique as being nearly pain free, potentially making it a more acceptable sampling method for diagnostic testing, particularly by individuals with needle and blood phobias. Due to the advantages offered by this ISF sampling technique, we envision that it can be readily employed in clinical settings to collect dermal ISF from individuals with known infections and medical conditions, leading to the identification of ISF-based biomarkers, including proteins, nucleic acids, and exosomes, associated with those diseases, which would advance progress in the use of dermal ISF for diagnostic testing.

EXPERIMENTAL PROCEDURES

Resource availability

Lead contact

Further information within reason should be directed to the lead contact, Peter B. Lillehoj (lillehoj@rice.edu).

Materials availability

This study did not generate new unique materials.

Data and code availability

This study did not generate/analyze any code. The data are available in the main text and supplemental information and are available from the lead contact upon reasonable request.

Fabrication of MN arrays

MN arrays were designed using NX software (Siemens, TX, USA) and printed in IP-Q resin using a Photonic Professional GT lithography system (NanoScribe, MA, USA). 3-mm-thick poly(methyl methacrylate) (PMMA) (McMaster Carr, IL, USA) was attached to the backside of the MN array for enhanced rigidity. MN arrays were fabricated via centrifugation-assisted replica molding (Figure S12). MN array master molds were made from polydimethylsiloxane (PDMS) (Sylgard 184, Dow, MI, USA). The PDMS was mixed at a 1:10 (curing agent-to-elastomer) ratio, degassed for 30 min, poured onto the MN array master, and heated in a convection oven at 80°C for 2 h. Cured PDMS was cut into individual molds using a razor blade and submerged in 70% isopropanol for 30 min. PDMS molds were dried overnight at room temperature before use. This process was used to create master molds for differently

sized MN arrays. MN array replicas were fabricated by pouring SU-8 2025 photoresist (Kayaku Advanced Materials, MA, USA) into the PDMS molds followed by centrifugation at 4,000 × *g* for 15 min. The molds were then placed under a 50 W UV (365 nm) lamp for 3 min for SU-8 polymerization. MN arrays were coated with 1.5 μm of parylene using a Labcoater 2 parylene deposition system (Specialty Coating Systems, IN, USA). MN arrays were characterized and imaged using a VHX-7000 optical microscope (Keyence Corporation, Osaka, Japan).

Mechanical testing of the MN arrays

The compression strength of the MN arrays was measured using a mechanical testing system (Instron, MA, USA). For each measurement, a single MN array was placed on the bottom plate of a 100 N load cell with the MN tips facing upward. The top plate was compressed from 0 to 90 N at a travel velocity of 0.5 mm min^{-1}. Force-displacement curves were obtained from three different MN arrays for each design, normalized in MATLAB (MathWorks, MA, USA) to set the initial position of the plate at zero displacement and plotted as the mean data ± SD in Microsoft Excel. Optical images of the MN arrays were taken before and after mechanical testing using a Keyence VHX-7000 microscope.

Skin penetration testing

MN arrays were tested on porcine skin to evaluate their skin penetration performance. Cadaver porcine skin from the abdominal area with hair, fat, and subcutaneous tissue removed was purchased from Animal Technologies (TX, USA). The skin was cut into 10 cm × 10 cm sections, vacuum sealed, and stored at −20°C. Prior to testing, a frozen skin section was thawed at room temperature and mounted onto foil-wrapped cardboard using safety pins. MNs tips were coated in blue ink using a fine-tip paintbrush (Zem Brush MFG, OH, USA), and the MN array was inserted into the skin section using an MN applicator (Micropoint Technologies, Singapore). To evaluate the durability of the MNs after repeated skin insertion, MN arrays were inserted into porcine skin 12 (20 × 20 array) or 36 (10 × 10 array) times, which were the maximum number of MN insertions to generate micropores for ISF extraction, using the MN applicator. Optical images of the MN arrays immediately following MN insertion (without post-cleaning) were obtained using a Keyence VHX-7000 microscope. To evaluate the pore size generated from the MNs, MN arrays were inserted into a flexible, wax membrane (composed of eight layers of Parafilm M) using the MN applicator. The Parafilm membrane was imaged using a Keyence VHX-7000 microscope, and pore size measurements were performed using the VHX-7000 microscope software (Ver 1.4.14.169). Results were presented as the average ± SD from five measurements for each MN length. To visualize the MN insertion wounds, histological analysis was performed on porcine skin sections following MN insertion. MNs were coated with Trypan blue (Sigma-Aldrich, USA) in glycerol (Sigma-Aldrich) solution using a fine-tip paintbrush, and the MN array was inserted into the skin section using an MN applicator. The skin sample was fixed in a 10% formalin solution (Sigma-Aldrich) for at least 48 h, transferred, and stored in a 70% ethanol solution. The sample was then embedded in paraffin (Sigma-Aldrich), dehydrated, sectioned, and stained with hematoxylin and eosin (H&E). Optical images of H&E-stained skin sections were captured using a Keyence VHX-7000 microscope.

Fabrication of the skin patch

The skin patch consists of a sticker and rigid plastic plate, both containing rectangular cutouts for the MN insertion sites. The sticker was fabricated from medical-grade, double-sided adhesive tape (3M Company, MN, USA), and the rigid plate was fabricated from 1.5-mm-thick PMMA (McMaster Carr). Double-sided, pressure-sensitive

adhesive tape (Adhesives Research, PA, USA) was attached to the top side of the plate. The sticker and rigid plate were designed using AutoCAD software (Autodesk, CA, USA) and cut using a CO_2 laser cutter (Universal Laser System, AZ, USA). The interior edges of the plate were sanded using a Dremel rotary tool to create smooth points of contact with the skin.

Sample collection from human volunteers

All procedures involving humans were conducted under the guidance and approval from the Rice University Institutional Review Board (IRB-FY2021-147). Criteria for participation was as follows: adults or Rice University students ages 18 or older with no blood clotting disorders (including hemophilia or factor II, V, VII, X, or XII deficiencies) or known skin allergies to medical adhesives. Potential participants were provided with informed consent to participate in the study. Participants were explained the entirety of the sample collection process prior to beginning the study. Informed consent of all participating subjects was obtained.

Twenty-eight adults were recruited for the study. ISF collection was carried out by first cleaning the participant's forearm using an alcohol prep pad (Fisher Healthcare, MA, USA) and attaching the skin patch sticker. The MN array was then applied to the skin two or three times at the MN insertion sites using an MN applicator. Immediately following MN insertion, the plastic plate was attached to the sticker followed by the attachment of a vacuum cup (Hansol Medical, South Korea). After ~3 min, vacuum pressure (−44 kPa) was generated inside the cup using a hand pump (Hansol Medical) and maintained for 20 min. The vacuum cup was then removed and the extracted ISF was collected using capillary tubes (Thermo Fisher Scientific, MA, USA, and Drummond Scientific Company, PA, USA). The skin patch was gently removed from the skin using an adhesive remover pad (Torbot Group, RI, USA), and the sampling site was cleaned using a fresh alcohol prep pad. After the study, participants were asked to complete a questionnaire rating the perceived pain levels associated with different steps of the sample collection procedure. The collected ISF sample was transferred to a low-bind microcentrifuge tube (Eppendorf, Hamburg, Germany), incubated at room temperature for 1 h, and centrifuged at 10,000 × g for 10 min. The supernatant was transferred to a new low-bind microcentrifuge tube, snap frozen in liquid N_2 for 5 min, and stored at −80°C until analysis.

Blood samples were obtained via fingerstick using a lancing device (Bayer Microlet) and 30G lancets (CareTouch). Blood was collected in capillary tubes (Thermo Fisher Scientific), transferred to a low-bind microcentrifuge tube, and incubated for 1 h at room temperature. The tube was then centrifuged at 10,000 × g for 10 min. Separated serum was transferred to a new low-bind microcentrifuge tube, snap frozen in liquid N_2 for 5 min, and stored at −80°C until analysis.

Proteomic analysis

Dermal ISF and plasma samples were first processed using a High Select Depletion Spin Column (Thermo Fisher Scientific, A36369) to remove abundant proteins. Samples were prepared for LC-MS/MS analysis by adjusting the sample solution to a final concentration of 5% sodium dodecyl sulfate and tetraethylammonium bromide (TEAB, 50 mM, pH 7.55, 25 μL). The samples were then centrifuged at 17,000 × g for 10 min to remove debris. The supernatant was transferred to a clean tube, and proteins were reduced by making TCEP (20 mM, Thermo Fisher Scientific, 77720) and incubated at 65°C for 30 min. The sample was cooled to room temperature, and iodoacetamide acid (0.5 M, 1 μL) was added and allowed to react for 20 min in the dark. Next, phosphoric acid (12%, 2.75 μL) was added to the protein solution,

and binding buffer (90% methanol, 100 mM TEAB, final pH 7.1, 165 μL) was then added to the solution. The resulting solution was added to an S-Trap spin column (Protifi, Fairport, NY) and passed through the column using a benchtop centrifuge (30-s spin at 4,000 × *g*). The spin column was washed with 400 μL of binding buffer (90% methanol, 100 mM TEAB, pH 7.55) and centrifuged. This process was repeated two more times. Trypsin was added to the protein mixture at a ratio of 1:25 in TEAB (50 mM, pH 8) and incubated at 37°C for 4 h. Peptides were eluted with TEAB (50 mM, 80 μL) followed by formic acid (0.2%, 80 μL) and finally acetonitrile (50%, 80 μL). The combined peptide solution was then dried in a SpeedVac and resuspended in acetonitrile (2%), formic acid (0.1%), and water (97.9%) and placed in an autosampler vial.

Peptide mixtures were analyzed by LC-MS/MS using a nanoflow LC chromatography system (UltiMate 3000 RSLCnano, Thermo Scientific, San Jose, CA), coupled online to a Thermo Orbitrap Eclipse mass spectrometer (Thermo Fisher Scientific) through a nanospray ion source coupled with a high-field asymmetric waveform ion mobility spectrometry (FAIMS) Pro device (Thermo Fisher Scientific) with Instrument Control Software (version 3.4). FAIMS separations were performed at standard resolution with the following settings: inner and outer electrode temperature = 100°C; FAIMS gas flow = 0 L min^{-1}, compensation voltages (CVs): −35, −55, and −75 with 1.3-s cycle times per CV. A direct injection method was used. MS1 mass spectra were acquired using a resolution setting of 120,000 (at 200 m z^{-1}), scanning from 400 to 1,600 m z^{-1}. Peptides were selected for MS/MS by data-dependent acquisition. Selected peptides were fragmented using higher energy collisional dissociation with a setting of 30% normalized collision energy, and peptide fragments were detected in the Orbitrap Eclipse ion trap using a Turbo scan rate. The analytical column was an Aurora capillary LC column (75 μm × 25 cm, 1.6 μm) obtained from Ion Opticks (Fitzroy, Vic, Australia). After equilibrating the column in 97% solvent A (0.1% formic acid in water) and 3% solvent B (0.1% formic acid in acetonitrile), the samples (2 μL in solvent A) were injected at 450 nL min^{-1} for 5 min when the flow was lowered to 300 nL min^{-1}. Peptides were eluted from the C18 column using a mobile phase gradient as follows: 3%–6%, 5–5.1 min; 6%–26% B, 5.1–125 min; 26%–40% B, 125–137 min; 40%–90% B, 137–140 min; isocratic at 90% B, 140–141 min; 90%–5%, 141–142 at 450 nL min^{-1}; isocratic at 5%, 142–142.5 min; 5%–95% 142.5–143 min; isocratic at 95% B, 143–144 min; 95%–5% B 144–145 min; and isocratic at 3% B until 160 min.

Protein identification

Tandem mass spectra were extracted, and the charge state was deconvoluted by a Proteome Discoverer (Thermo Fisher, version 2.5). Deisotoping was not performed. All MS/MS spectra were searched against a Uniprot human database and a common contaminant database (cRAP, version 03-29-2016) using SEQUEST. Searches were performed with a parent ion tolerance of 5 ppm and a fragment ion tolerance of 0.60 Da. Trypsin is specified as the enzyme, allowing for two missed cleavages. Fixed modification of carbamidomethyl (C) and variable modifications of oxidation (M) and deamidation were specified in SEQUEST. The protein false discovery rate [FDR] validator node was used to estimate to calculate experimental q values, and a cutoff of 1.0% FDR was applied.

Proteomic identification results were further analyzed using two online biomarker databases, OncoMX[49] and BIONDA.[50] In each database, the accession number was entered, and the results were filtered when necessary. In OncoMX, data were reported as an FDA or an EDRN biomarker. In BIONDA, data were reported as the associated disease. All results were manually cross-checked for accuracy.

Absolute protein concentration measurements

Absolute concentrations of protein in blood and ISF samples were determined using a Pierce Coomassie (Bradford) Protein Assay Kit (Thermo Fisher Scientific). Paired dermal ISF and fingerstick blood samples from five volunteers were analyzed in triplicate. The samples were diluted 100× in PBS. Bovine serum albumin standards were diluted to 1,500, 1,000, 750, 500, 250, 125, 25, and 0 μg mL^{-1} with PBS. The standard microplate protocol with a working range of 125–1,500 μg mL^{-1} was used. Briefly, each standard or sample (5 μL) was mixed with Coomassie reagent (250 μL) in a microplate well (Thermo Fisher Scientific) and then incubated for 10 min at room temperature. Absorbance measurements were read at 595 nm using a Biotek Epoch microplate spectrophotometer (Agilent, CA, USA).

SARS-CoV-2 neutralizing antibody detection in dermal ISF

Paired dermal ISF and fingerstick blood samples from volunteers were analyzed for the presence of SARS-CoV-2 neutralizing antibody using a lateral flow antibody detection device (RayBiotech, USA). Tests were performed according to the manufacturer's instructions using freshly collected fingerstick blood or dermal ISF. Images of the test results were captured using a smartphone camera. SARS-CoV-2 neutralizing antibody concentrations were measured in dermal ISF using a SARS-CoV-2 Surrogate Virus Neutralization test kit (GenScript USA, USA). Briefly, dermal ISF samples and SARS-CoV-2 neutralizing antibody standard (GenScript USA, USA) with concentrations of 0, 9.375, 18.75, 37.5, 75, 150, 300, and 600 ng mL^{-1} were prepared, and each sample (10 μL) was diluted with sample dilution buffer (90 μL). Diluted samples were mixed with diluted horseradish peroxide (HRP) conjugated recombinant SARS-CoV-2 receptor binding domain (RBD) fragment (HRP-RBD) solution with a 1:1 volume ratio. Each mixture (100 μL) was added to the corresponding well. The plate was covered and incubated at 37°C for 15 min, and wells were rinsed four times with wash buffer. 3,3′,5,5′-Tetramethylbenzidine solution (100 μL) was added to each well, and the plate was incubated in the dark at room temperature for 15 min. Stop solution (50 μL) was added to each well to quench the reaction. The colorimetric signal was read immediately using a Biotek Epoch microplate spectrophotometer at a wavelength of 450 nm. Duplicate measurements were run for each sample.

Statistics

Statistical analysis was performed using GraphPad Software (Prism 9.5 version). Statistical differences were determined using a two-tailed Student's t test, one-way ANOVA with Tukey's post hoc, or two-way ANOVA with Tukey's post hoc, according to the number of groups being analyzed. The type of test was indicated in conjunction with each p value when reported throughout the manuscript. $p < 0.05$ was considered statistically significant in all cases.

SUPPLEMENTAL INFORMATION

Supplemental information can be found online at https://doi.org/10.1016/j.xcrp.2024.101975.

ACKNOWLEDGMENTS

We acknowledge the Shared Equipment Authority of Rice University for the use of the cleanroom facilities to fabricate the MN arrays. We thank Marquise Bell and Dr. Daniel Preston for allowing us to use their mechanical testing machine and for providing assistance with mechanical testing of the MN arrays. We thank Lize Chen for his assistance in filtering the proteomic data. We thank Lee Palmer for

processing the samples for proteomics analysis. E.C.W. provided material based upon work supported by the National Science Foundation Graduate Research Fellowship (1842494). The LC-MS/MS analysis was conducted at The University of Texas Medical Branch's Mass Spectrometry Facility, which is supported by the Cancer Prevention Research Institute of Texas grant no. RP190682. This research was funded by the Wellcome Trust (215826/Z/19/Z).

AUTHOR CONTRIBUTIONS

X.J. and E.C.W. contributed equally. Conceptualization: P.B.L. Data curation: X.J., E.C.W., A.O.B., and W.K.R. Formal analysis: X.J., E.C.W., A.O.B., and W.K.R. Investigation: X.J., E.C.W., A.O.B., and P.B.L. Methodology: X.J., E.C.W., and P.B.L. Validation: X.J. and E.C.W. Visualization: X.J., E.C.W., and P.B.L. Writing – original draft: X.J., E.C.W., A.O.B., W.K.R., and P.B.L. Writing – review & editing: X.J., E.C.W., and P.B.L. Funding acquisition: P.B.L. Project administration: P.B.L. Supervision: P.B.L.

DECLARATION OF INTERESTS

X.J. is affiliated with Open Avenues Foundation and Spear Bio Inc. A.O.B. is affiliated with AbCellera Biologics Inc. X.J., E.C.W., and P.B.L. are co-inventors of a provisional patent application filed by Rice University on the subject of this work.

Received: February 15, 2024
Revised: April 5, 2024
Accepted: April 19, 2024
Published: May 9, 2024

REFERENCES

1. Bogers, J.P.A.M., Bui, H., Herruer, M., and Cohen, D. (2015). Capillary compared to venous blood sampling in clozapine treatment: Patients' and healthcare practitioners' experiences with a point-of-care device. Eur. Neuropsychopharmacol *25*, 319–324. https://doi.org/10.1016/j.euroneuro.2014.11.022.

2. Lassandro, G., Amoruso, A., Palladino, V., Valeria, V., and Giordano, P. (2021). The risk of venipuncture in newborn with severe hemophilia : Case report of a large elbow hemorrhage and literature review of compartment syndrome. Hematol. Rep. *13*. https://doi.org/10.4081/hr.2021.8967.

3. Piorino, F., Patterson, A.T., and Styczynski, M.P. (2022). Low-cost, point-of-care biomarker quantification. Curr. Opin. Biotechnol. *76*, 102738. https://doi.org/10.1016/j.copbio.2022.102738.

4. Sim, D., Brothers, M.C., Slocik, J.M., Islam, A.E., Maruyama, B., Grigsby, C.C., Naik, R.R., and Kim, S.S. (2022). Biomarkers and Detection Platforms for Human Health and Performance Monitoring. Adv. Sci. *9*, 1–29. https://doi.org/10.1002/advs.202104426.

5. Dutkiewicz, E.P., and Urban, P.L. (2016). Quantitative mass spectrometry of unconventional human biological matrices. Philos. Trans. A Math. Phys. Eng. Sci. *374*, 20150380. https://doi.org/10.1098/rsta.2015.0380.

6. Aukland, K., and Nicolaysen, G. (1981). Interstitial fluid volume: local regulatory mechanisms. Physiol. Rev. *61*, 556–643. https://doi.org/10.1152/physrev.1981.61.3.556.

7. Mccrudden, M.T.C., Mcalister, E., Courtenay, A.J., González-Vázquez, P., Singh, T.R.R., and Donnelly, R.F. (2015). Microneedle applications in improving skin appearance. Exp. Dermatol. *24*, 561–566. https://doi.org/10.1111/exd.12723.

8. Samant, P.P., and Prausnitz, M.R. (2018). Mechanisms of sampling interstitial fluid from skin using a microneedle patch. Proc. Natl. Acad. Sci. USA *115*, 4583–4588. https://doi.org/10.1073/pnas.1716772115.

9. Tran, B.Q., Miller, P.R., Taylor, R.M., Boyd, G., Mach, P.M., Rosenzweig, C.N., Baca, J.T., Polsky, R., and Glaros, T. (2018). Proteomic Characterization of Dermal Interstitial Fluid Extracted Using a Novel Microneedle-Assisted Technique. J. Proteome Res. *17*, 479–485. https://doi.org/10.1021/acs.jproteome.7b00642.

10. Müller, A.C., Breitwieser, F.P., Fischer, H., Schuster, C., Brandt, O., Colinge, J., Superti-Furga, G., Stingl, G., Elbe-Bürger, A., and Bennett, K.L. (2012). A comparative proteomic study of human skin suction blister fluid from healthy individuals using immunodepletion and iTRAQ labeling. J. Proteome Res. *11*, 3715–3727. https://doi.org/10.1021/pr3002035.

11. Kool, J., Reubsaet, L., Wesseldijk, F., Maravilha, R.T., Pinkse, M.W., D'Santos, C.S., van Hilten, J.J., Zijlstra, F.J., and Heck, A.J.R. (2007). Suction blister fluid as potential body fluid for biomarker proteins. Proteomics *7*, 3638–3650. https://doi.org/10.1002/pmic.200600938.

12. Ribet, F., Bendes, A., Fredolini, C., Dobielewski, M., Böttcher, M., Beck, O., Schwenk, J.M., Stemme, G., and Roxhed, N. (2023). Microneedle Patch for Painless Intradermal Collection of Interstitial Fluid Enabling Multianalyte Measurement of Small Molecules, SARS-CoV-2 Antibodies, and Protein Profiling. Adv. Healthcare Mater. *12*, e2202564. https://doi.org/10.1002/adhm.202202564.

13. Miller, P.R., Taylor, R.M., Tran, B.Q., Boyd, G., Glaros, T., Chavez, V.H., Krishnakumar, R., Sinha, A., Poorey, K., Williams, K.P., et al. (2018). Extraction and biomolecular analysis of dermal interstitial fluid collected with hollow microneedles. Commun. Biol. *1*, 173. https://doi.org/10.1038/s42003-018-0170-z.

14. Jina, A., Tierney, M.J., Tamada, J.A., McGill, S., Desai, S., Chua, B., Chang, A., and Christiansen, M. (2014). Design, development, and evaluation of a novel microneedle array-based continuous glucose monitor. J. Diabetes Sci. Technol. *8*, 483–487. https://doi.org/10.1177/1932296814526191.

15. Samant, P.P., Niedzwiecki, M.M., Raviele, N., Tran, V., Mena-Lapaix, J., Walker, D.I., Felner, E.I., Jones, D.P., Mil-ler, G.W., and Prausnitz, M.R. (2020). Sampling interstitial fluid from human skin using a microneedle patch. Sci. Transl. Med. *12*, eaaw0285–16 *In Press*.

16. Ribet, F., Dobielewski, M., Böttcher, M., Beck, O., Stemme, G., and Roxhed, N. (2020).

CellPress
OPEN ACCESS

Minimally invasive and volume-metered extraction of interstitial fluid: bloodless point-of-care sampling for bioanalyte detection. Sens. Bio-Sensing Res. https://kth.diva-portal.org/smash/record.jsf?pid=diva2%3A1388020&dswid=5775.

17. Friedel, M., Thompson, I.A.P., Kasting, G., Polsky, R., Cunningham, D., Soh, H.T., and Heikenfeld, J. (2023). Opportunities and challenges in the diagnostic utility of dermal interstitial fluid. Nat. Biomed. Eng. *7*, 1541–1555. https://doi.org/10.1038/s41551-022-00998-9.

18. Saifullah, K.M., and Faraji Rad, Z. (2023). Sampling Dermal Interstitial Fluid Using Microneedles: A Review of Recent Developments in Sampling Methods and Microneedle-Based Biosensors. Adv. Mater. Interfac. *10*. https://doi.org/10.1002/admi.202201763.

19. Krogstad, A.L., Jansson, P.A., Gisslén, P., and Lönnroth, P. (1996). Microdialysis methodology for the measurement of dermal interstitial fluid in humans. Br. J. Dermatol. *134*, 1005–1012. https://doi.org/10.1111/j.1365-2133.1996.tb07934.x.

20. Pieber, T.R., Wach, P., Mangge, H., Ellmerer, M., Schaupp, L., Brunner, G.A., Wutte, A., Sendlhofer, G., Trajanoski, Z., and Skrabal, F. (2008). Direct Access to Interstitial Fluid in Adipose Tissuein Humans by Use of Open-Flow Microperfusion, pp. 401–408.

21. Venugopal, M., Feuvrel, K.E., Mongin, D., Bambot, S., Faupel, M., Panangadan, A., Talukder, A., and Pidva, R. (2008). Clinical evaluation of a novel interstitial fluid sensor system for remote continuous alcohol monitoring. IEEE Sensor. J. *8*, 71–80. https://doi.org/10.1109/JSEN.2007.912544.

22. Sieg, A., Guy, R.H., and Delgado-Charro, M.B. (2004). Noninvasive glucose monitoring by reverse iontophoresis in vivo: Application of the internal standard concept. Clin. Chem. *50*, 1383–1390. https://doi.org/10.1373/clinchem.2004.032862.

23. Kolluru, C., Williams, M., Chae, J., and Prausnitz, M.R. (2019). Recruitment and Collection of Dermal Interstitial Fluid Using a Microneedle Patch. Adv. Healthcare Mater. *8*, e1801262. https://doi.org/10.1002/adhm.201801262.

24. Waghule, T., Singhvi, G., Dubey, S.K., Pandey, M.M., Gupta, G., Singh, M., and Dua, K. (2019). Microneedles: A smart approach and increasing potential for transdermal drug delivery system. Biomed. Pharmacother. *109*, 1249–1258. https://doi.org/10.1016/j.biopha.2018.10.078.

25. Xu, J., Xu, D., Xuan, X., and He, H. (2021). Advances of Microneedles in Biomedical Applications. Molecules *26*. https://doi.org/10.3390/molecules26195912.

26. Al-Kasasbeh, R., Brady, A.J., Courtenay, A.J., Larrañeta, E., McCrudden, M.T.C., O'Kane, D., Liggett, S., and Donnelly, R.F. (2020). Evaluation of the clinical impact of repeat application of hydrogel-forming microneedle array patches. Drug Deliv. Transl. Res. *10*, 690–705. https://doi.org/10.1007/s13346-020-00727-2.

27. Mukerjee, E.V., Collins, S.D., Isseroff, R.R., and Smith, R.L. (2004). Microneedle array for transdermal biological fluid extraction and in situ analysis. Sensors Actuators, A Phys. *114*, 267–275. https://doi.org/10.1016/j.sna.2003.11.008.

28. Chen, Z., and Lee, J.B. (2021). Biocompatibility of SU-8 and Its Biomedical Device Applications. Micromachines *12*, 794. https://doi.org/10.3390/mi12070794.

29. Kuppusami, S., and Oskouei, R.H. (2015). Parylene Coatings in Medical Devices and Implants: A Review. ujbe. *3*, 9–14. https://doi.org/10.13189/ujbe.2015.030201.

30. Davis, S.P., Landis, B.J., Adams, Z.H., Allen, M.G., and Prausnitz, M.R. (2004). Insertion of microneedles into skin: Measurement and prediction of insertion force and needle fracture force. J. Biomech. *37*, 1155–1163. https://doi.org/10.1016/j.jbiomech.2003.12.010.

31. Schmook, F.P., Meingassner, J.G., and Billich, A. (2001). Comparison of human skin or epidermis models with human and animal skin in in-vitro percutaneous absorption. Int. J. Pharm. *215*, 51–56. https://doi.org/10.1016/S0378-5173(00)00665-7.

32. Samant, P.P., Niedzwiecki, M.M., Raviele, N., Tran, V., Mena-Lapaix, J., Walker, D.I., Felner, E.I., Jones, D.P., Miller, G.W., and Prausnitz, M.R. (2020). Sampling interstitial fluid from human skin using a microneedle patch. Sci. Transl. Med. *12*, eaaw0285–16. https://doi.org/10.1126/SCITRANSLMED.AAW0285.

33. You, J.S., Gelfanova, V., Knierman, M.D., Witzmann, F.A., Wang, M., and Hale, J.E. (2005). The impact of blood contamination on the proteome of cerebrospinal fluid. Proteomics *5*, 290–296. https://doi.org/10.1002/pmic.200400889.

34. Grady, M., Pineau, M., Pynes, M.K., Katz, L.B., and Ginsberg, B. (2014). A clinical evaluation of routine blood sampling practices in patients with diabetes: Impact on fingerstick blood volume and pain. J. Diabetes Sci. Technol. *8*, 691–698. https://doi.org/10.1177/1932296814533172.

35. Baker, L.B. (2017). Sweating Rate and Sweat Sodium Concentration in Athletes: A Review of Methodology and Intra/Interindividual Variability. Sports Med. *47*, 111–128. https://doi.org/10.1007/s40279-017-0691-5.

36. Ghezzi, E.M., Lange, L.A., and Ship, J.A. (2000). Determination of variation of stimulated salivary flow rates. J. Dent. Res. *79*, 1874–1878. https://doi.org/10.1177/00220345000790111001.

37. Rozenfeld, E., and Kalichman, L. (2016). New is the well-forgotten old: The use of dry cupping in musculoskeletal medicine. J. Bodyw. Mov. Ther. *20*, 173–178. https://doi.org/10.1016/j.jbmt.2015.11.009.

38. Mankoski, A. (1995). Mankoski Pain Scale. https://www.valis.com/andi/painscale.html.

39. Dalton, E.L., and Velasquez, B.J. (2017). Cupping Therapy: An Alternative Method of Treating Pain. Public Health Open J. *2*, 59–63. https://doi.org/10.17140/phoj-2-122.

40. Svedman, C., Yu, B.B., Ryan, T.J., and Svensson, H. (2002). Plasma proteins in a standardised skin mini-erosion (I): permeability changes as a function of time. BMC Dermatol. *2*. https://doi.org/10.1186/1471-5945-2-3.

41. Reed, R.K., and Rubin, K. (2010). Transcapillary exchange: Role and importance of the interstitial fluid pressure and the extracellular matrix. Cardiovasc. Res. *87*, 211–217. https://doi.org/10.1093/cvr/cvq143.

42. Leeman, M., Choi, J., Hansson, S., Storm, M.U., and Nilsson, L. (2018). Proteins and antibodies in serum, plasma, and whole blood—size characterization using asymmetrical flow field-flow fractionation (AF4). Anal. Bioanal. Chem. *410*, 4867–4873. https://doi.org/10.1007/s00216-018-1127-2.

43. Sloop, C.H., Dory, L., and Roheim, P.S. (1987). Interstitial fluid lipoproteins. J. Lipid Res. *28*, 225–237. https://doi.org/10.1016/s0022-2275(20)38701-0.

44. Kalra, A., and Lowe, A. (2016). Mechanical Behaviour of Skin: A Review. J. Mater. Sci. Eng. *5*. https://doi.org/10.4172/2169-0022.1000254.

45. Kim, S., Lee, M.S., Yang, H.S., and Jung, J.H. (2021). Enhanced extraction of skin interstitial fluid using a 3D printed device enabling tilted microneedle penetration. Sci. Rep. *11*, 14018. https://doi.org/10.1038/s41598-021-93235-3.

46. Makvandi, P., Kirkby, M., Hutton, A.R.J., Shabani, M., Yiu, C.K.Y., Baghbantaraghdari, Z., Jamaledin, R., Carlotti, M., Mazzolai, B., Mattoli, V., et al. (2021). Engineering Microneedle Patches for Improved Penetration: Analysis, Skin Models and Factors Affecting Needle Insertion. Nano-Micro Lett. *13*, 1–41. https://doi.org/10.1007/s40820-021-00611-9.

47. Kim, J., Park, S., Nam, G., Choi, Y., Woo, S., and Yoon, S.H. (2018). Bioinspired microneedle insertion for deep and precise skin penetration with low force: Why the application of mechanophysical stimuli should be considered. J. Mech. Behav. Biomed. Mater. *78*, 480–490. https://doi.org/10.1016/j.jmbbm.2017.12.006.

48. Larrañeta, E., Moore, J., Vicente-Pérez, E.M., González-Vázquez, P., Lutton, R., Woolfson, A.D., and Donnelly, R.F. (2014). A proposed model membrane and test method for microneedle insertion studies. Int. J. Pharm. *472*, 65–73. https://doi.org/10.1016/j.ijpharm.2014.05.042.

49. Dingerdissen, H.M., Bastian, F., Vijay-Shanker, K., Robinson-Rechavi, M., Bell, A., Gogate, N., Gupta, S., Holmes, E., Kahsay, R., Keeney, J., et al. (2020). OncoMX: A Knowledgebase for Exploring Cancer Biomarkers in the Context of Related Cancer and Healthy Data. JCO Clin. Cancer Inform. *4*, 210–220. https://doi.org/10.1200/cci.19.00117.

50. Turewicz, M., Frericks-Zipper, A., Stepath, M., Schork, K., Ramesh, S., Marcus, K., and Eisenacher, M. (2021). BIONDA: a free database for a fast information on published biomarkers. Bioinform. Adv. *1*, vbab015–3. https://doi.org/10.1093/bioadv/vbab015.

Cell Reports Physical Science, Volume *5*

Supplemental information

Microneedle-based sampling of dermal interstitial fluid using a vacuum-assisted skin patch

Xue Jiang, Elizabeth C. Wilkirson, Aaron O. Bailey, William K. Russell, and Peter B. Lillehoj

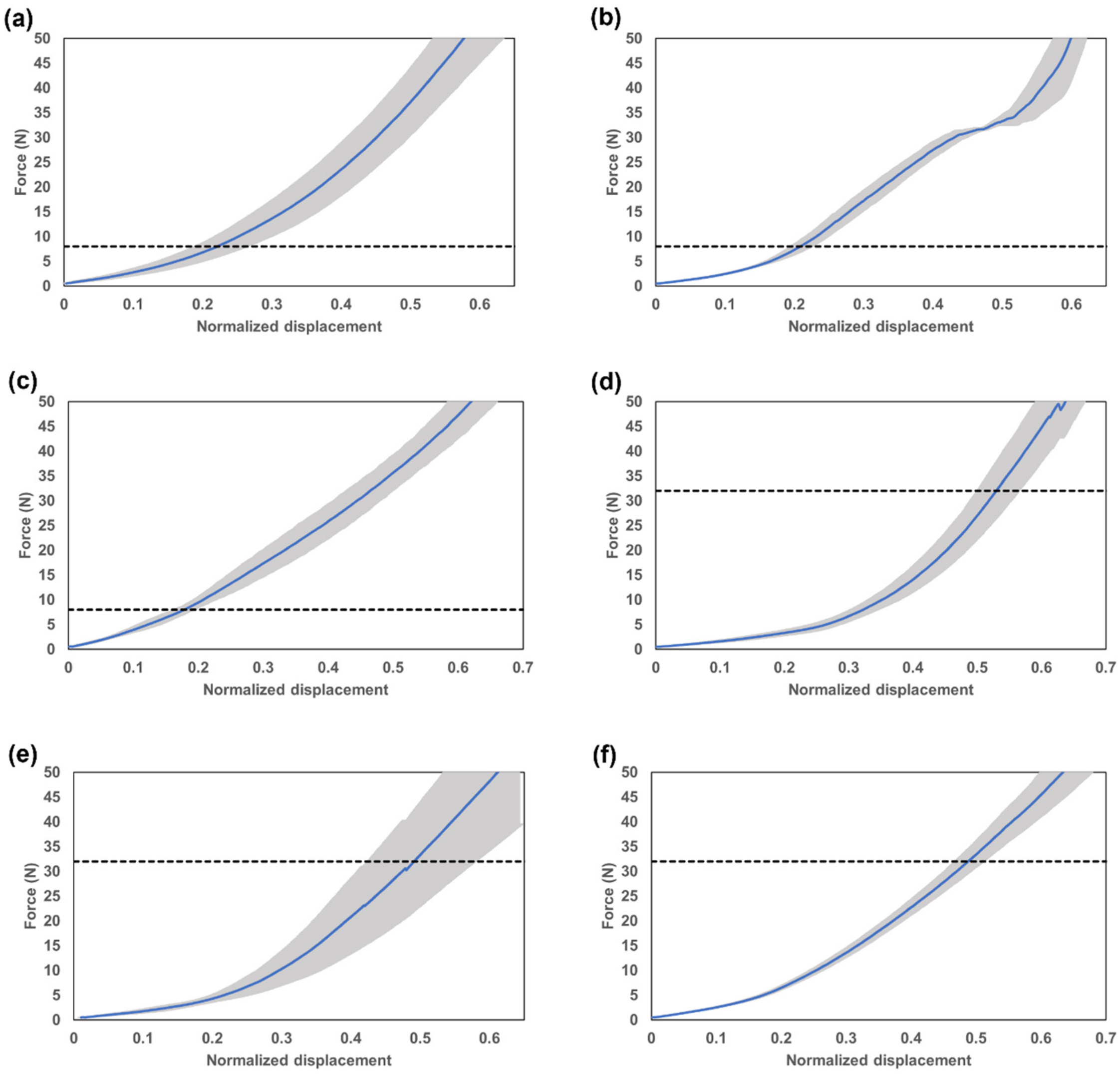

Figure S1. Mechanical strength of MN arrays. Force vs. displacement curves for 10 × 10 MN arrays with needle lengths of (a) 450 μm, (b) 600 μm or (c) 750 μm, and (d) 20 × 20 MN arrays with needle lengths of 450 μm, (e) 600 μm, or (f) 750 μm. Solid lines represent the mean from 3 separate measurements using new MN arrays. Shaded region represents the standard deviation. Horizontal dotted lines represent the force required to penetrate human skin.

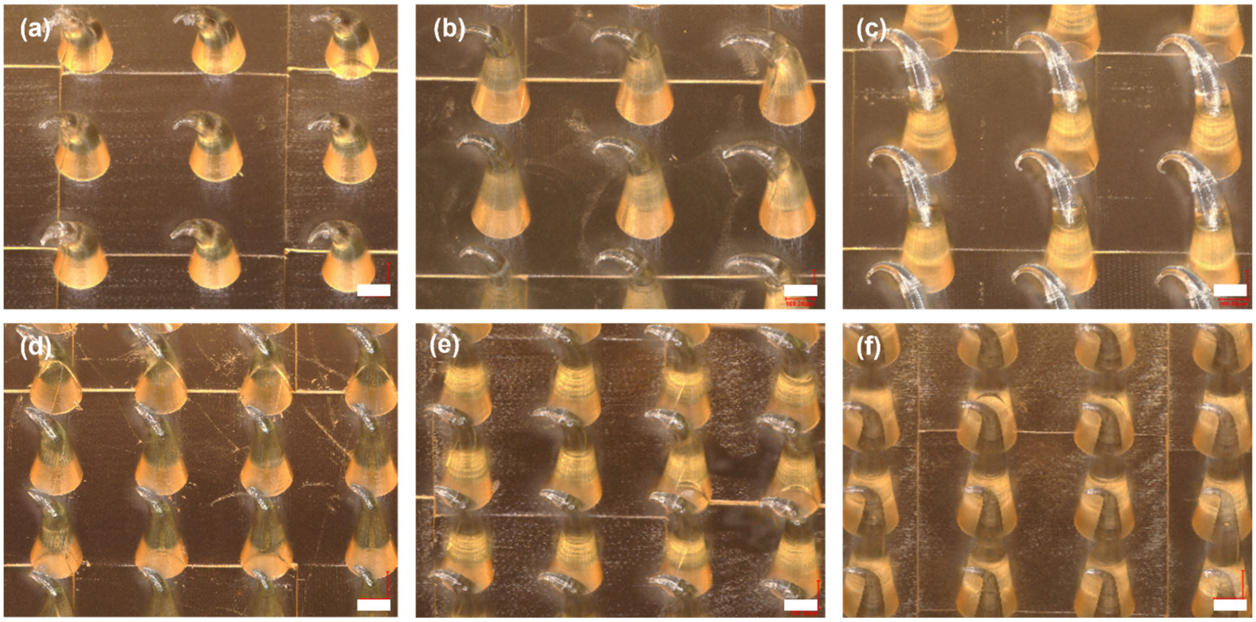

Figure S2. Compression testing of MN arrays at maximum load. Optical micrographs of 10 × 10 MN arrays with needle lengths of (a) 450 μm, (b) 600 μm, or (c) 750 μm, and (d) 20 × 20 MN arrays with needle lengths of 450 μm, (e) 600 μm, or (f) 750 μm. Scale bars, 100 μm.

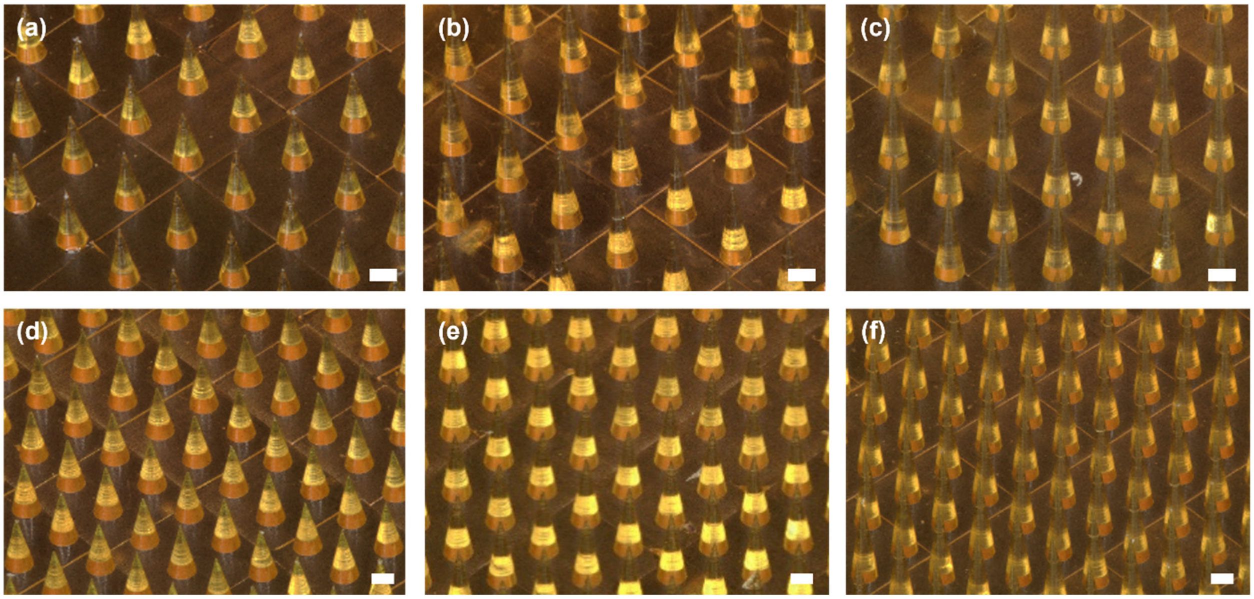

Figure S3. MN integrity after repeated skin penetration. Optical micrographs of 10 × 10 MN arrays with needle lengths of (a) 450 μm, (b) 600 μm, or (c) 750 μm after 36 insertions in porcine skin, and (d) 20 × 20 MN arrays with needle lengths of 450 μm, (e) 600 μm, or (f) 750 μm after 12 insertions. Scale bars, 1000 μm. Insets show close-up views of the MNs. Scale bars, 100 μm.

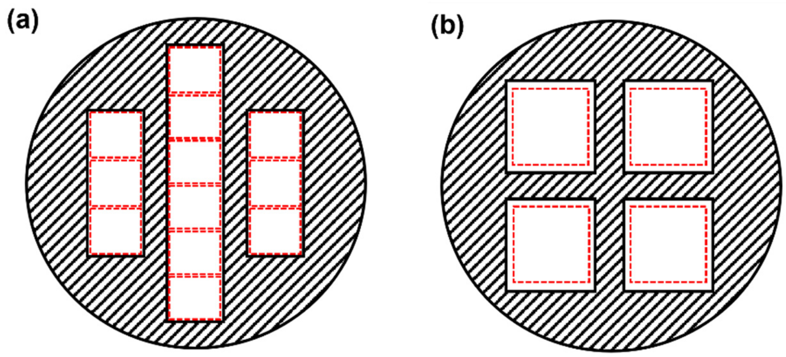

Figure S4. Designs of the skin patch sticker. MN array application sites outlined in red for the (a) 10 × 10 MN array and (b) 20 × 20 MN array.

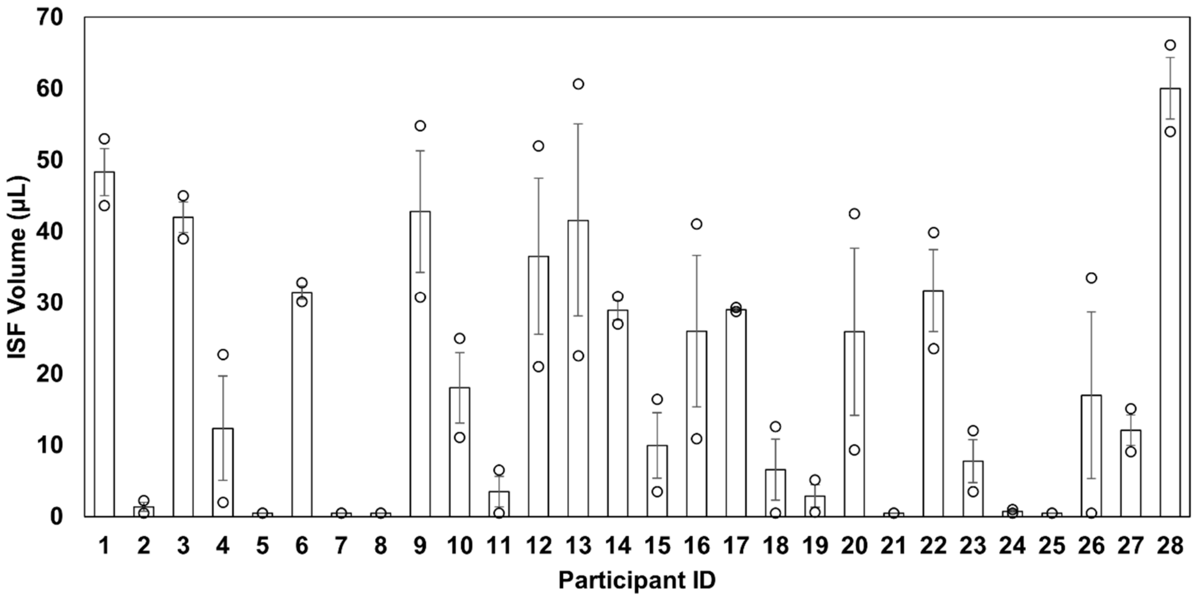

Figure S5. ISF collection volume from each participant. Each dot represents the volume of dermal ISF collected from one sample collection. Bars represent the average collection volume sampled from each participant from two independent sample collections.

Cell Reports
Physical Science

CellPress

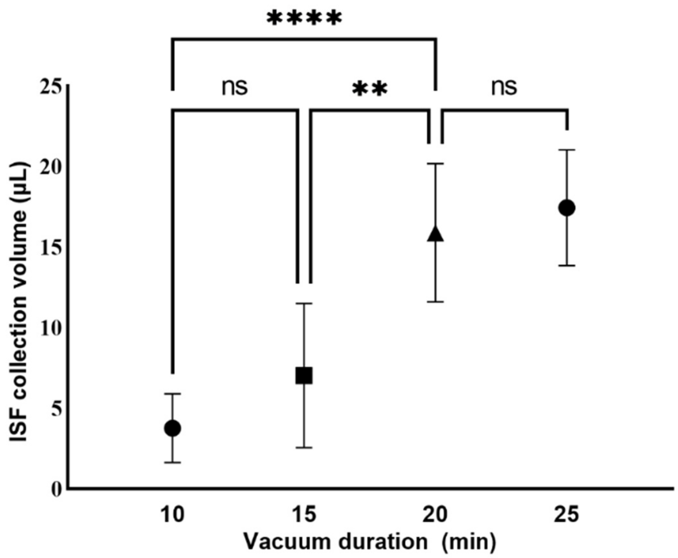

Figure S6. Influence of vacuum duration on ISF collection volume. ISF collection volume obtained using varying durations of applied vacuum. Experiments were performed using 20 × 20 MN arrays with needle lengths of 450 µm or 600 µm and three MN insertions per application site. Each data point represents the mean ± SD obtained from six independent sample collections (n=6). Significance was determined by one-way ANOVA with Tukey's post hoc (**p=0.0028, ****p<0.0001).

Cell Reports
Physical Science

(a) **PAIN SCALE**

0 — Pain free

1 — Very minor annoyance-occasional minor twinges

2 — Minor annoyance-occasional

3 — Annoying enough to be distracting

4 — Can be ignored if you are really involved in your work, but still distracting,

5 — Can't be ignored for more than 30 minutes.

6 — Can't be ignored for any length of time, but you can still go to work and participate in social activities.

7 — Make it difficult to concentrate, interferes with sleep, you can still function with effort

8 — Physical activity severely limited. You can read and converse with effort. Nausea and dizziness may occur.

9 — Unable to speak, crying out or moaning uncontrollable- pain makes you pass out

10 — Unconscious. Pain makes you pass out.

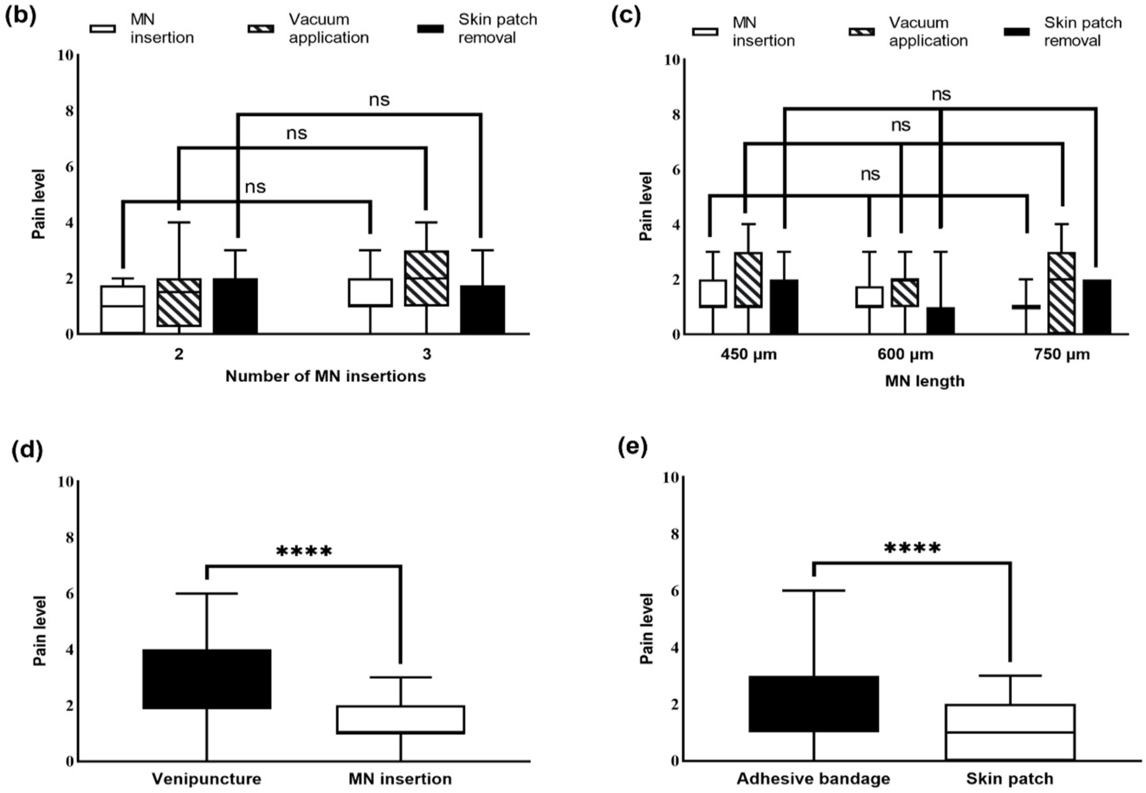

Figure S7. Self-reported pain levels associated with the ISF sampling procedure. (a) Pain level rating scale. (b) Participants' responses for pain level associated with different number of MN insertions per application site. Statistics acquired by Student's *t* test (ns=P>0.05). (c) Participants' responses for pain level associated with different MN lengths. Significance was determined by one-way ANOVA with Tukey's post hoc (ns=P>0.05). For comparison purposes, participants also rated their perceived pain levels during a standard venipuncture procedure and standard bandage removal. Participants' responses for comparing pain level between (d) MN insertion vs. venipuncture and (d) removal of the skin patch vs. removal of a standard adhesive bandage. Significance was determined with Student's *t* test (****p<0.0001). Each bar represents the mean ± min/max of responses from participants (n=26).

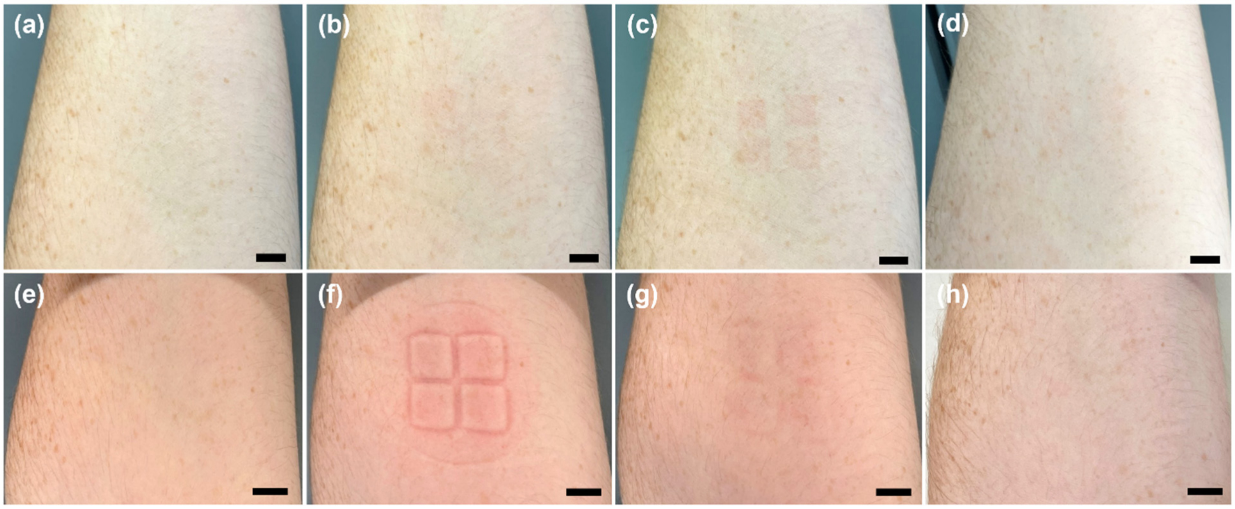

Figure S8. Effects of ISF sampling on the skin. Photographs of the sampling site from one participant (a) before MN insertion, (b) immediately after MN insertion, (c) 2 hr after MN insertion, and (d) 24 hr after MN insertion without vacuum application. Photographs of the sampling site from one participant (e) before ISF sampling, (f) immediately after ISF sampling, (g) 2 hr after ISF sampling, and (h) 24 hr after ISF sampling. Scale bars, 10 mm.

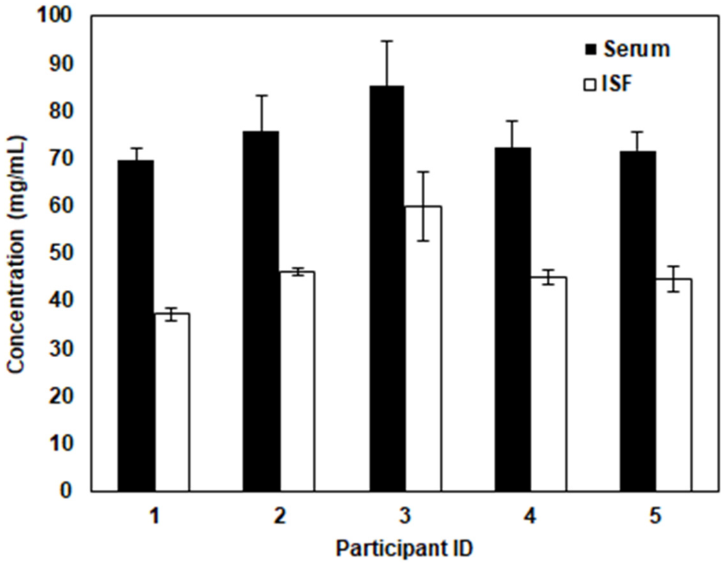

Figure S9. Absolution protein concentration in paired dermal ISF and blood serum samples from five volunteers. Each bar represents the mean ± SD of three measurements (n=5 participant triplicates).

Cell Reports
Physical Science
CellPress

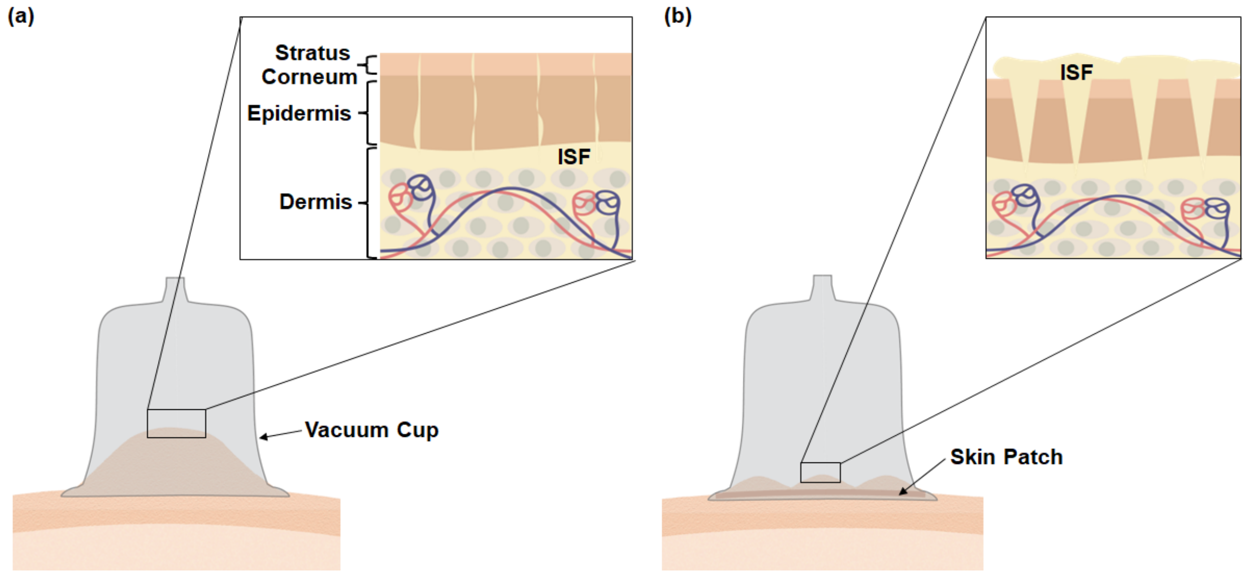

Figure S10. Schematic illustrations depicting ISF sampling with and without the skin patch. (a) Without the skin patch, the skin deforms excessively when suction is applied, causing the micropores to close. (b) With the skin patch, the skin is made taut when suction is applied, which induces the opening of the micropores, facilitating ISF extraction.

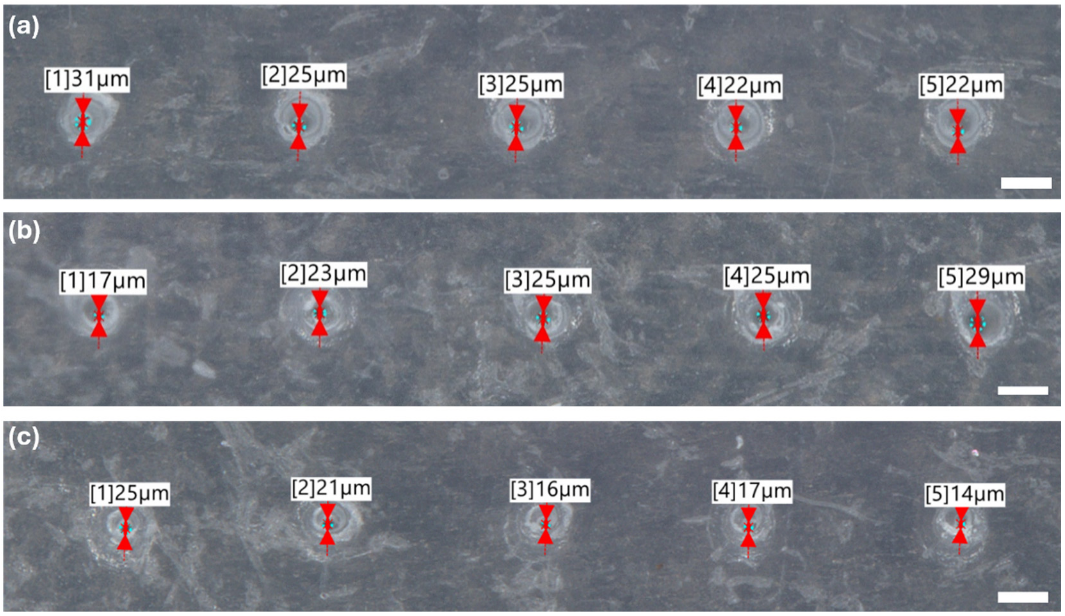

Figure S11. Micropore size as a function of MNs length. The insertion of MNs into a wax-based membrane model resulted in pore sizes of (a) 25 ± 3.67 μm for 450 μm-long MNs, (b) 23.8 ± 4.38 μm for 600 μm-long MNs, and (c) 18.6 ± 4.39 μm for 750 μm-long MNs. Each set of data represents the mean ± SD of 5 pores (n=5). Scale bars, 100 μm.

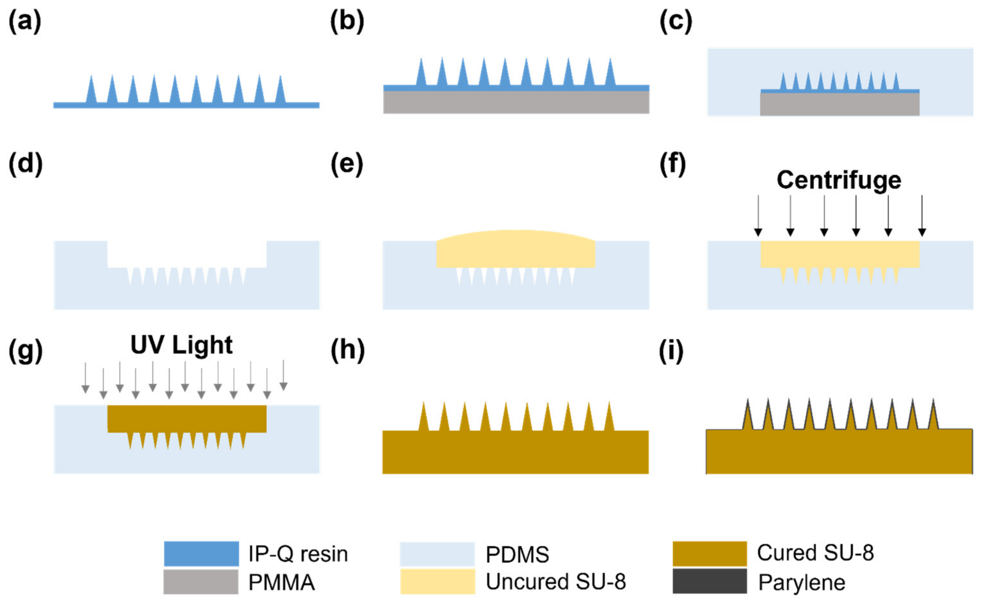

Figure S12. MN array fabrication process. (a) MN array master is fabricated using a NanoScribe 3D printer. (b) A 3 mm-thick PMMA substrate is attached to the backside of the MN array. (c)-(d) PDMS master mold is made via replica molding. (e) SU-8 is dropcasted on the PDMS mold. (f) Mold is centrifuged at 4,000 g for 15 min. (g) Exposure to 365 nm UV light for 3 min. (h) Polymerized SU-8 MN array is removed from PDMS mold. (i) MN array is coated in a layer of parylene.

Table S1. Demographics of the 28 participants enrolled in the ISF collection study.

Age (years)	
Mean ± SD	24.1 ± 3.8
Median	23.5
**Sex**	
Male	13
Female	15
**Ethnicity**	
Caucasian	7
African American	2
Hispanic	2
Asian	14
Multiple	3

Cell Reports Physical Science

CellPress

Table S2. Demographics of the 5 participants enrolled in the ISF collection study whose samples were analyzed using LC-MS/MS.

**Age (years)**	
Mean ± SD	29.6 ± 5.1
Median	28
**Sex**	
Male	2
Female	3
**Ethnicity**	
Caucasian	1
Asian	3
Prefer not to answer	1

Cell Reports
Physical Science

CellPress

Table S3. Comparison of MN-based techniques for sampling ISF from human skin.

Type of MN	Equipment Needed for ISF Extraction	ISF Extraction Time	Average Collection Volume (μL)	Reference
Hydrogel	None	6 hr	-	[S1]
Hollow	None	5 min	1.1	[S2]
Hollow	None	15-20 min	< 1	[S3]
Hollow	Capillary tubes	1-2 hr	16	[S4, S5]
Solid	Electrical vacuum pump	20 min	2.3 ± 2.1	[S6]
Solid	Electrical vacuum pump	20 min	3.4 ± 3.2	[S7]
Solid	Vacuum cup and hand pump	20 min	20.8 ± 19.4	This work

Supplemental References

S1. Al-Kasasbeh, R., Brady, A.J., Courtenay, A.J., Larrañeta, E., McCrudden, M.T.C., O'Kane, D., Liggett, S., and Donnelly, R.F. (2020). Evaluation of the clinical impact of repeat application of hydrogel-forming microneedle array patches. Drug Deliv. Transl. Res. *10*, 690–705. 10.1007/s13346-020-00727-2.

S2. Ribet, F., Dobielewski, M., Böttcher, M., Beck, O., Stemme, G., and Roxhed, N. (2020). Minimally invasive and volume-metered extraction of interstitial fluid: bloodless point-of-care sampling for bioanalyte detection. Sens. Bio-Sensing Res.

S3. Mukerjee, E. V., Collins, S.D., Isseroff, R.R., and Smith, R.L. (2004). Microneedle array for transdermal biological fluid extraction and in situ analysis. Sensors Actuators, A Phys. *114*, 267–275. 10.1016/j.sna.2003.11.008.

S4. Tran, B.Q., Miller, P.R., Taylor, R.M., Boyd, G., Mach, P.M., Rosenzweig, C.N., Baca, J.T., Polsky, R., and Glaros, T. (2018). Proteomic Characterization of Dermal Interstitial Fluid Extracted Using a Novel Microneedle-Assisted Technique. J. Proteome Res. *17*, 479–485. 10.1021/acs.jproteome.7b00642.

S5. Miller, P.R., Taylor, R.M., Tran, B.Q., Boyd, G., Glaros, T., Chavez, V.H., Krishnakumar, R., Sinha, A., Poorey, K., Williams, K.P., et al. (2018). Extraction and biomolecular analysis of dermal interstitial fluid collected with hollow microneedles. Commun. Biol. *1*. 10.1038/s42003-018-0170-z.

S6. Samant, P.P., and Prausnitz, M.R. (2018). Mechanisms of sampling interstitial fluid from skin using a microneedle patch. Proc. Natl. Acad. Sci. U. S. A. *115*, 4583–4588. 10.1073/pnas.1716772115.

S7. Samant, P., Niedzwiecki, M. M., Raviele, N., Tran, V., Lapaix, J. M., Walker, D. I., Felner, E. I., Jones, D. P., Mil-ler, G. W. & Prausnitz, M.R. (2020). Sampling interstitial fluid from human skin using a microneedle patch. Sci. Transl. Med. *In Press*, 1–16.
